# In-depth genetic and molecular characterization of diaphanous related formin 2 (*DIAPH2*) and its role in the inner ear

**DOI:** 10.1371/journal.pone.0273586

**Published:** 2023-01-23

**Authors:** Chiara Chiereghin, Michela Robusto, Morag A. Lewis, Susana Caetano, Valentina Massa, Pierangela Castorina, Umberto Ambrosetti, Karen P. Steel, Stefano Duga, Rosanna Asselta, Giulia Soldà

**Affiliations:** 1 IRCCS Humanitas Research Hospital, Rozzano, Milan, Italy; 2 Experimental Therapeutics Program, IFOM ETS -The AIRC Institute of Molecular Oncology, Milan, Italy; 3 Wolfson Centre for Age-Related Diseases, King’s College London, London, United Kingdom; 4 Dipartimento di Scienze della Salute, Università degli Studi di Milano, Milano, Italy; 5 Casa di Cura Igea, Milano, Italy; 6 Dipartimento di Scienze Cliniche e di Comunità, Università degli Studi di Milano and Fondazione IRCCS Cà Granda Ospedale Maggiore Policlinico, UO Audiologia, Milano, Italy; 7 Humanitas University, Department of Biomedical Sciences, Pieve Emanuele, Milan, Italy; University of Iowa, UNITED STATES

## Abstract

Diaphanous related formins are regulatory cytoskeletal protein involved in actin elongation and microtubule stabilization. In humans, defects in two of the three diaphanous genes (*DIAPH1* and *DIAPH3*) have been associated with different types of hearing loss. Here, we investigate the role of the third member of the family, *DIAPH2*, in nonsyndromic hearing loss, prompted by the identification, by exome sequencing, of a predicted pathogenic missense variant in *DIAPH2*. This variant occurs at a conserved site and segregated with nonsyndromic X-linked hearing loss in an Italian family. Our immunohistochemical studies indicated that the mouse ortholog protein Diaph2 is expressed during development in the cochlea, specifically in the actin-rich stereocilia of the sensory outer hair cells. *In-vitro* studies showed a functional impairment of the mutant DIAPH2 protein upon RhoA-dependent activation. Finally, *Diaph2* knock-out and knock-in mice were generated by CRISPR/Cas9 technology and auditory brainstem response measurements performed at 4, 8 and 14 weeks. However, no hearing impairment was detected. Our findings indicate that *DIAPH2* may play a role in the inner ear; further studies are however needed to clarify the contribution of *DIAPH2* to deafness.

## Introduction

Nonsyndromic hearing loss (NSHL) accounts for approximately 70% of genetic deafness cases. NSHL is characterized by an extreme genetic heterogeneity with more than 120 responsible genes identified (Hereditary Hearing Loss Homepage: https://hereditaryhearingloss.org/, last accessed on 27 May 2022) and possibly others yet to be found. Indeed, it is estimated that over 3% of genes (as many as 1,000 genes) may have a function in hearing [1, 2]. Deafness-causing genes belong to broad functional categories that contribute to the numerous molecular processes fundamental for hearing, such as mechanotransduction and cochlear ion homeostasis. For instance, several genes encoding proteins involved in the structure and function of sensory hair cells are associated with NSHL, including cytoskeletal proteins. Among these, *diaphanous*-related formins (DRFs) are important regulators of actin nucleation and microtubule dynamics [3, 4]. These multi-domain proteins are active as dimers and can be functionally divided into two parts. The C-terminal half is required for actin assembly and includes three domains: i) the FH1 (formin homology 1) domain, which binds profilin/monomeric actin; ii) the FH2 (formin homology 2) domain, which can bind microfilaments (F-actin) and microtubules; and iii) the DAD (*diaphanous* autoregulatory domain), which can interact with the N-terminal portion. The N-terminal half has mainly a regulatory function and contains the GTPase binding domain (GBD) and the *diaphanous* inhibitory domain (DID) [5]. In addition, a dimerization domain (DD) and a coiled coil (CC) domain are needed for dimerization [3]. DRFs are kept in an inactive conformation by intramolecular interaction between the C-terminal DAD and the N-terminal DID, which is also involved in the interaction with Rho family GTPases [6, 7]. As a consequence of this partial overlap between the DAD and Rho binding sites, the binding of Rho GTPases induces the transition to the active conformation with the release of the autoinhibitory domain, thus stimulating actin nucleation [8].

In vertebrates, three DRF genes (*DIAPH1*, *DIAPH2* and *DIAPH3*) are the orthologs of the *Drosophila melanogaster diaphanous* (dia) gene, which has important roles in fertility and hearing in the fly. Indeed, mutant *dia* alleles cause both sterility and impaired response to sound [9, 10]. In humans, *DIAPH1* and *DIAPH3* have been associated with different types of hearing loss (HL) [3]: heterozygous mutations in *DIAPH1* are responsible for autosomal dominant NSHL with or without thrombocytopenia (locus *DFNA1*; Mendelian Inheritance in Man, MIM #124900) [11, 12], whereas overexpression of *DIAPH3* –resulting from a mutation in the 5’ untranslated region (UTR) of the gene—causes auditory neuropathy, autosomal dominant, 1 (*AUNA1*; MIM #609129) [10]. Concerning *DIAPH2*, located on chromosome Xq21.33, until now it has only been linked to premature ovarian failure 2 (*POF2A*; MIM #300511). In particular, in a mother and daughter affected by POF, a balanced translocation t(X,12)(q21,p13) was found to disrupt the *DIAPH2* gene [13].

Here, we identified a missense variant in *DIAPH2* segregating with NSHL in an Italian family and we tested its causal role, as well as the function of the *DIAPH2* gene in normal hearing function.

## Materials and methods

### Subjects enrolled in the study

The study was reviewed and approved by the Ethical Committee of the Fondazione IRCCS Cà Granda Ospedale Maggiore Policlinico of Milan (date of approval 20/09/2011) and complies to the rules indicated in the Declaration of Helsinki on research involving human subjects. All subjects signed a written informed consent for inclusion before they participated in the study. For subjects younger than 18 years, the informed consent was obtained from their parents. Samples were anonymized and sensitive data were treated according to the Italian legislation and the European General Data Protection Regulation (GDPR).

**Fig 1:**
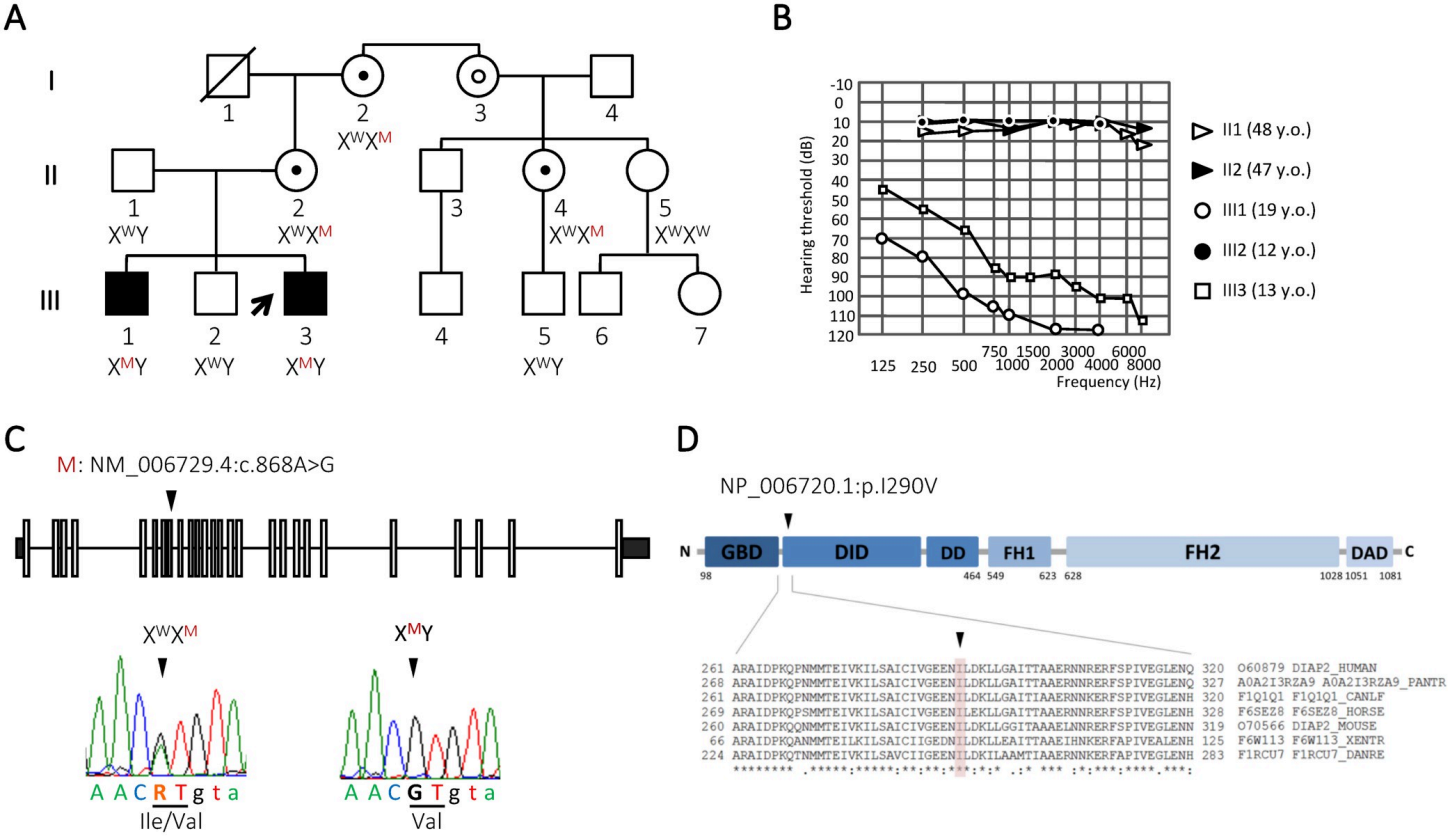
Identification of a missense variant in the candidate *DIAPH2* gene. (**A**) Pedigree of an Italian family with nonsyndromic hearing loss, showing the segregation of the *DIAPH2* variant (NM_006729.4:c.868A>G) within the family. The genotypes of available individuals are indicated below the corresponding symbol and the proband is indicated by a black arrow. W: wild-type allele (A), M: mutant allele (G). ○: obligate carrier, ●: carrier of the variant confirmed by Sanger sequencing. (**B**) Pure-tone air-conduction thresholds. For each subject the threshold average for the right and left ear and the age at audiometric evaluation is shown. The two affected siblings III1 and III3 are indicated by empty circles and squares, respectively. Their brother (III2, black circles), mother (II2, black triangles), and father (II1, empty triangles) show normal hearing. (**C**) Schematic representation of *DIAPH2* gene, where exons are indicated by rectangles and introns by black lines. Electropherograms showing the sequence surrounding the mutated nucleotide in the proband (right) and his mother (heterozygous carrier, left). The position of the identified variant is indicated by an arrowhead. R: A or G. Exonic nucleotides are indicated with uppercase, while intronic nucleotides are indicated with lowercase letters. (**D**) Schematic representation of DIAPH2 protein and amino acid sequence alignments of DIAPH2 orthologs in the region surrounding the mutant residue (p.I290). The position of the p.I290V is indicated by an arrowhead. Protein sequences were retrieved from UniProt, and alignments were generated with Clustal Omega. The affected amino acid residue is shadowed. Identical amino acids are marked by an asterisk, while partially conserved residues are indicated by a colon. GBD: GTPase-binding domain, DID: *diaphanous* inhibitory domain, DD: dimerization domain, FH1: formin homology 1, FH2: formin homology 2, DAD: *diaphanous* autoregulatory domain, N: N-terminus, C: C-terminus.

One Italian family affected by NSHL was recruited (Fig 1). Clinical history ruled out environmental factors as cause of deafness and physical examination did not reveal any evidence of dysmorphic features. An in-depth clinical analysis to exclude syndromic forms of deafness was performed on both affected children (III1 and III3), including: ECG, renal echography, eye examination with funduscopic examination, electroretinography and urine test. No family history for other diseases associated with variations in *DIAPH2* (i.e. *POF2A*) was reported. Evoked potentials were also tested using both click-evoked and tone burst auditory brainstem response (ABR). Sixteen probands from families with suspected X-linked HL were also recruited. All available subjects underwent ear, nose and throat examinations, and pure-tone audiometry, in accordance with International Standard Organization (ISO 8253-1-3) protocols. Average hearing thresholds in the range of 21–40 dB were defined as mild, 41–70 dB as moderate, 71–95 dB as severe, and >95 dB as profound deafness. All recruited individuals were previously screened for mutations in the gap-junction proteins connexin 26 and 30 (*GJB2*, *GJB6*), and the mitochondrial 12S rRNA (*MTRNR1*) genes. A cohort of 125 Italian subjects with no family history of hearing loss and normal auditory function verified by audiometry (mean age at enrolment 32±9) was included in the study. In addition, a population-specific control cohort was used, consisting of 3,541 individuals. Exome sequencing and data analysis were performed by the Broad Institute, as previously described [14].

### Exome sequencing

Exome sequencing was performed starting from 50 ng of blood-derived genomic DNA and using the Nextera Rapid Capture Exome Enrichment kit (Illumina, San Diego, CA), following the manufacturer’s instructions. Libraries were sequenced as paired-end 150-bp reads on a NextSeq500 platform (Illumina) at the Humanitas Genomic facility. Reads were aligned to hg19 reference genome using the BWA (Burrows-Wheeler Aligner 0.7.7) program [15], and coverage of known autosomal-recessive or X-linked NSHL genes was specifically assessed (S1 Table in S1 File). Variant calling and annotation were performed, respectively, with GATK (Genome Analysis Toolkit 1.6) [16] and Annovar (2016Feb01) software [17]. Nonsynonymous/splice-site single nucleotide variants (SNVs) and insertion-deletions (indels) were filtered against 1000 Genomes Project and the Genome Aggregation Database (gnomAD, v.2.1.1, https://gnomad.broadinstitute.org, last accessed on 27 May 2022), setting to 1% the threshold for the maximum minor allele frequency (MAF) [18, 19]. Assuming a recessive inheritance pattern, variants present at the homozygous/hemizygous or compound heterozygous state were prioritized. Then, only variants shared between the two affected brothers were selected as candidate pathogenic mutations (S2 Table in S1 File). Finally, the pathogenicity of candidate variant(s) was predicted using the Combined Annotation Dependent Depletion (CADD) software [20]. The frequency of the final prioritized variants (S2 Table in S1 File) was further checked in the ALFA dataset (ALFA: Allele Frequency Aggregator: https://www.ncbi.nlm.nih.gov/snp/docs/gsr/alfa/, last accessed on 26 June 2022) and in an in-house cohort of 3,541 Italian control exomes, to identify population-specific variants. A specific analysis pipeline was also applied to assess the potential functional impact of synonymous variants on protein function and/or splicing, by using a combination of 4 different tools: CADD [20]; DANN, a deep learning approach for annotating the pathogenicity of genetic variants, including synonymous ones [21]; dbscSNV [22], which analyzes all potential human SNVs within splicing consensus regions (comprising nucleotides from −3 to +8 at the 5’ splice site and from −12 to +2 at the 3’ splice site) and predict their potential of altering splicing; SpliceAI [23], a deep neural network that accurately predicts splice junctions from an arbitrary pre-mRNA transcript sequence, enabling precise prediction of genetic variants that cause cryptic splicing. Default thresholds suggested by the tool developers were used to prioritize potentially pathogenic synonymous variants.

Besides point mutations, copy number variant (CNV) analysis was performed directly on exome data, using EXCAVATOR2 [24, 25]. Default parameters and a window size of 50 kb were set for the analyses. The three siblings were used as test samples, and 5 exomes (3 males, 2 females) from other in-house NSHL families with a known cause of deafness (i.e. pathogenic single nucleotide variants in known deafness genes) were used as controls for CNV calling. Only CNVs shared between the two affected siblings were selected for further inspection (S3 Table in S1 File).

NSHL genes not adequately covered by exome sequencing (S1 Table in S1 File) as well as candidate variants were investigated by Sanger sequencing. Briefly, genomic regions of interest were PCR amplified using sets of primers designed based on the known GenBank sequence (S4 Table in S1 File), or previously published and validated [26, 27]. PCRs were performed on 10–20 ng of genomic DNA, following standard procedures. In some cases (i.e. STRC exons 1–19) a long-range PCR was performed followed by nested Sanger sequencing, as previously described [27]. Direct sequencing of PCR amplicons was performed on both strands using the BigDye Terminator Cycle Sequencing Ready Reaction Kit v.1.1 and an automated ABI-3500 DNA sequencer (Thermo Fisher Scientific, Waltham, MA, USA). The Variant Reporter software (Thermo Fisher Scientific) was used for variant detection.

### Expression vectors

The expression vector containing human *DIAPH2* coding sequence (pCMV-HA-hDIA2B) was kindly provided by the laboratory of Professor Marino Zerial [28]. The NM_006729.4:c.868A>G point variation was introduced by site-direct mutagenesis, using a specific primer pair (5’-GTTGGAGAAGAGAACgTTCTAGATAAACTTT-3’ and 5’-AAAGTTTATCTAGAAcGTTCTCTTCTCCAAC-3’, where the lowercase letter corresponds to the variant nucleotide) and the QuickChange Site-Directed Mutagenesis Kit (Agilent Technologies, Santa Clara, CA, USA). The plasmid encoding a constitutively active form of RhoA (pCDNA3.1-Myc-RhoA-G14V), used in DIAPH2 immunolocalization studies, was provided by the laboratory of Professor Taroh Iiri [29]. Recombinant plasmids were extracted with the PureYield Plasmid Midiprep System (Promega, Madison, WI, USA), and verified by sequencing.

### Cell cultures and transfections

Human embryonic kidney 293 (HEK293) cells were cultured in Dulbecco’s modified Eagle medium supplemented with 2 mM L-glutamine, 10% fetal bovine serum (FBS) and antibiotics (100 U/ml penicillin and 100 μg/ml streptomycin; Euroclone, Wetherby, UK) and grown at 37°C in a humidified atmosphere of 5% CO_2_ and 95% air, according to standard procedures.

For immunofluorescence assays, 2.5*10^5^ HEK293 cells were seeded on 22x22 mm glass coverslips and transfected with 1 μg of plasmid encoding either the wild-type or the mutant protein under study, using the Jet-PRIME reagent (Euroclone). For immunolocalization of the DIAPH2 protein, an equimolar quantity of the pcDNA3.1_Myc_RhoA-G14V vector was also co-transfected.

### Cell immunofluorescence experiments

Cells seeded on coverslips were fixed 48 hours after transfection using 4% paraformaldehyde for 10 minutes at room temperature. Cells were washed twice for 15 minutes in 100 mM glycine and permeabilized/blocked for 30 minutes in GDB (gelatin dilution buffer: 0.1% gelatin, 0.3% Triton X-100, 450 mM NaCl, 20 mM phosphate buffer pH 7.4). Coverslips were washed in HS (high salt: 500 mM NaCl, 20 mM phosphate buffer pH 7.4) buffer and incubated for 2 hours with primary antibodies diluted in GDB (1:75: Anti-HA probe, sc-7392, Santa-Cruz Biotechnology, Dallas, TX, USA, 1:100: Anti-Myc tag, ab9106, Abcam, Cambridge, UK). The cells were then washed 3 times in HS buffer and incubated 1 hour with fluorophore-conjugated secondary antibodies (Dylight 549 anti-mouse, 115-505-174, Jackson ImmunoResearch Laboratories, West Grove, PA, USA, Alexa Fluor 633 anti-rabbit, A21072, Molecular Probes, Eugene, OR, USA both 1:200 diluted). After 3 washes in HS buffer, cell nuclei were counterstained with DAPI (4’,6-Diamidino-2-Phenylindole, Molecular Probes) for 5 minutes and cells were washed once in HS and LS (low salt: 150 mM NaCl, 10 mM phosphate buffer pH 7.4) buffers and bidistilled water. Coverslips were mounted with FluorPreserve reagent (Merck Millipore, Burlington, MA, USA). For actin staining, cells were labelled with ActinGreen 488 ReadyProbes Reagent (Molecular Probes) before mounting, following the manufacturer’s instructions. Confocal images were acquired using a 63x HC PL APO 63x oil-immersion objective (N.A. 1.40, Leica, Wetzlar, Germany) with a Leica TCS SP8 confocal microscope at a resolution of 1 airy unit. Identical gain, offset, exposure, and laser-power settings were applied to wild-type and mutant conditions in every experiment. Images were analyzed with the Fiji (Fiji Is Just ImageJ) software [30]. The mean length of membrane protrusions was measured in 20 wt and 20 mut HEK293 cells using Fiji. Statistical analyses were performed with GraphPad Prism 8.0 (GraphPad software, San Diego, California USA, www.graphpad.com). An unpaired t-test was applied to calculate statistical significance of the observed differences in the length of membrane protrusions between cells expressing wild-type or mutant DIAPH2 protein.

### Immunohistochemistry on wax sections

Immunohistochemical studies were performed to evaluate the expression of the Diaph2 protein in the mouse inner ear. For all experiments E14.5, E16.5, P5, P7 and P14 mice of the C57BL/6 strain and E18.5 and P0 mice with a C3HeB/FeJ genetic background were used. For each age, a minimum of three mice were tested. Mouse experiments were performed as previously described [31] and under the regulations, licenses and local ethical review from Queen Mary University of London following UK Home Office Animals (Scientific Procedures) Act 1986. Briefly, females were bred with males of the same strain at the end of the daily light cycle and checked for vaginal plugs the following morning. The day of the vaginal plug was considered as embryonic age 0.5 dpc (days post coitum). Pregnant females were then sacrificed by cervical dislocation at desired gestational age for collecting embryos.

Formalin-fixed paraffin-embedded wild-type mouse heads were sectioned sagittally at 8 μm thick using the Leica RM2255 microtome, mounted on Superfrost Plus slides (Thermo Fisher Scientific) and incubated at 42°C overnight. The Ventana Discovery machine and reagents (all from Roche, Basel, Switzerland) were used for immunohistochemistry, following the manufacturer’s instructions. The slides were incubated for 1 hour with the goat anti-Diaph2 antibody (1:50, sc-10892, Santa Cruz Biotechnology) and subsequently incubated for 16 minutes with the Biotin-SP AffiniPure Donkey Anti-Goat IgG (H+L) secondary antibodies (1:100, 705-065-147, Jackson ImmunoResearch). Primary and secondary antibodies were diluted in a phosphate-buffered saline (PBS) solution containing 10% FBS, 2% bovine serum albumin, 0.1% Triton-X100, and 10 mM sodium azide. After incubation with SA-HRP (streptavidin-horseradish peroxidase) the immunohistochemistry reaction was revealed using DAB (3,3’-Diaminobenzidine). Finally, slides were counterstained with hematoxylin. After staining, the slides were manually dehydrated using ascending ethanol concentrations (70%, 85%, 100%) and subsequently washed in acetone and xylene before mounting with Eukitt mounting medium (Sigma-Aldrich, Saint-Louis, MO, USA) and 24x50 mm coverslips. Brightfield images of the sections were acquired using a Zeiss Axioskop microscope (Zeiss, Oberkochen, Germany) connected with the Axiovision 3.0 software.

### Knock-out and knock-in *Diaph2* mouse models

Mouse studies were performed in accordance with the UK Home Office regulations and the UK Animals (Scientific Procedures) Act 1986 (ASPA) under UK Home Office licenses, and the study was approved by the King’s College London Ethical Review Committee. Mice were culled using methods approved under these licenses to minimize any possibility of suffering.

The *Diaph2*^*em2Kcl*^ knock-out mice and the *Diaph2*^*em3Kcl*^ knock-in mice were generated by the Genome Editing & Embryology Core (King’s College London, London, UK) using the Clustered Regularly Interspaced Short Palindromic Repeats (CRISPR)-Cas9 technology. Both mice were generated on a pure C57BL/6J genetic background. *Diaph2*^*em2Kcl*^ mice display a 19-bp deletion (NM_172493.2:c.855_873del) resulting in a frameshift and hence creating a premature stop codon in exon 9. *Diaph2*^*em3Kcl*^ mice display the missense variant NM_172493.2:c.877A>G, NP_766081.1:p.I293V in exon 8, corresponding to the NM_006729.4:c.868A>G, NP_006720.1:p.I290V variant identified in the Italian NSHL family. Of note, three additional synonymous variants were introduced (NM_172493.2:c.852T>C, NM_172493.2:c.855T>A, NM_172493.2:c.867G>A) in order to destroy the guide RNA (gRNA) binding and Protospacer adjacent motif (PAM) site—thus preventing the re-cutting of Cas9 after successful integration of the oligo donor.

### ABR recordings

ABR measurements were performed following the protocol described in [32]. A series of broadband click stimuli (0.01 msec duration) and tone pips (3, 6, 12, 18, 24, 30, 36, 42 kHz, 5 msec duration, 1 msec raise/fall time) were presented free-field over a range of intensity levels from 0 to 95 dB sound pressure level (SPL) in 5-dB steps. Responses were detected using subcutaneous pin electrodes placed behind each ear and at the vertex. The averaged ABR waveforms, resulting from the average of responses to 256 stimuli presented at a rate of 42.6/sec, were visually analyzed and thresholds established as the lowest sound intensity giving a detectable ABR response.

ABR waveforms were analyzed by comparing waveforms from a group of mice of the same age and genotype for each frequency/click at the same stimulus level above threshold (dB HL).

## Results

### Exome sequencing identifies a candidate variant within the *DIAPH2* gene

An Italian family with two NSHL-affected siblings was recruited; the pedigree is compatible with recessive (autosomal or X-linked) inheritance (Fig 1A). The proband (III3) is a 17-year-old male with a 25-year-old deaf brother (III1) and a 22-year-old normal-hearing brother (III2). Both affected siblings have pre-lingual bilateral HL that appeared more severe in the middle and high frequencies. HL in III3 was moderate in the low frequencies and severe-to-profound in middle-high frequencies (Fig 1B); the tympanogram was normal for both ears and acoustic (stapedius) reflexes were present bilaterally. In III1 HL was severe in the low and profound in the middle-high frequencies (Fig 1B). Click-evoked ABR were tested in III3 when he was 4 years old, and the wave V thresholds were set at 70 dB and 75 dB for the right and left ear, respectively. Tone burst ABR at 450–500 Hz detected a threshold at 55 dB bilaterally (S1 Fig in S1 File). ABRs were absent in the older sibling (III1) when aged 12. Genetic screening of the gap junction beta subunit genes *GJB2* and *GJB6*, which encode the predominant connexin isoforms in the cochlea and represent the most commonly mutated genes in NSHL patients [33], was previously performed. The NM_004004.5:c.457G>A (NP_003995.2:p.V153I) variant in *GJB2* (dbSNP ID rs111033186) was detected in the heterozygous state in one of the deaf children (III3), the normal hearing child, and their mother (II2). This missense variant, located in *GJB2* exon 2, is annotated as benign in ClinVar (accession number VCV000044754.5). Indeed, it is reported to occur with a frequency of 5.3% in the South Asian Population (1,642/3,0610 chromosomes) according to gnomAD (last accessed on 27 May 2022), including 52 homozygous individuals. Moreover, the variant did not segregate with HL in two previously reported pedigrees [34, 35], and was found in the homozygous state or the heterozygous state together with a pathogenic *GJB2* mutation in normal-hearing individuals [36, 37]. The three siblings (III1, III2 and III3) were hence subjected to exome sequencing. On average, we generated 8.4 Gb of high-quality sequence data/exome, with a mean coverage of the target region of 76X.

Specific analysis of known autosomal-recessive or X-linked NSHL genes, (76 and 5, respectively; Hereditary Hearing Loss Homepage: https://hereditaryhearingloss.org/, last accessed on 26 June 2022) did not detect bi-allelic candidate pathogenic variants, after verifying coverage of the entire coding sequence in exome data (S1 Table in S1 File). Coverage of multiple exons was missing for only two genes (*STRC*, *OTOA*), which are well known to have mapping issues due to the presence of segmental duplications and/or pseudogenes. Nonetheless, we screened by Sanger sequencing all the missing exons/genes and verified the absence of mutations. In addition, CNV analysis on exome data did not evidence any alteration within known deafness loci (S3 Table in S1 File). Subsequently, we analyzed all potential pathogenic variants detected by exome sequencing and shared between the affected siblings, but not the normal hearing one. Only nonsynonymous/splicing variants with MAF ≤ 1% in 1000 Genomes Project [19] /GnomAD exome databases and a CADD [20] score ≥ 20 were selected, and an autosomal recessive or X-linked mode of inheritance was hypothesized. Moreover, we excluded genes located within ENCODE Blacklist regions (e.g. *MUC3A*), as they likely represent next-generation sequencing artifacts, with high signal independent from cell line, experiment, or the specific phenotype under study [38]. A total of 12 variants in 5 genes (*FAM136A*; *FAM8A1*; *ESRRA*; *MXRA5*; *DIAPH2*) were found (Table 1, S2 Table in S1 File). Further inspection of these variants highlighted that most of them had a frequency in the general population greater than 1%, as annotated in the ALPHA database, but were not found in GnomAD because they are flagged as low-quality variants (S2 Table in S1 File).

**Table 1:** Prioritization of variants identified by exome sequencing.

Filter	III1	III2	III3
**NS/SS/I**	13,963	13,801	14,239
**1000 Genomes/gnomAD MAF≤0.01%**	2,058	2,042	2,133
**Hom/Hemi/compound Het**	197	194	201
**Only shared between III1 and III3**	22		-
**CADD phred score ≥20**	12		-

NS: non-synonymous variant, SS: splice-site variant, I: indel, MAF: Minor Allele Frequency, Hom: homozygous variant, Hemi: hemizygous variant, Het: heterozygous variant, CADD: Combined Annotation Dependent Depletion.

Besides the standard variant prioritization pipeline, a further analysis was performed specifically on synonymous variants, which are commonly discarded as likely non-pathogenic, although in some cases might impact on gene function, for example by altering splicing. Four different tools were used to assess the potential functional impact of synonymous variants, either by estimating their likelihood of being deleterious with CADD and DANN [20, 21], or by evaluating their predicted effect on splicing with dbscSNV and SpliceAI [22, 23]. As shown in Table 2, by combining the results from different predictors we found 7 synonymous variants (in 7 different genes) that were shared between the two affected siblings and might have a functional impact (S2 Table in S1 File). None of them was located in a known deafness-causing gene. As all the 7 variants were present in both siblings at the heterozygous state, we looked for a second shared variant in the same gene, but none was found.

**Table 2:** Prioritization of synonymous variants based on their predicted functional impact.

Filter	Prediction tool			
	CADD	DANN	dbscSNV	spliceAI
**Rare synonymous variants (MAF<1%)**	1,269	1,269	1,269	1,269
**Variant above threshold** ^a^	7	46	2	4 (3 AG, 1 DL)
**Variant shared between affected siblings; state**	0	6^b^; het	1^c^; het	0
**Second shared variant in the same gene**	na	none	none	na

^a^tool-specific thresholds: CADD Phred score ≥ 20; DANN score ≥ 0.93; dbscSNV score ≥ 0.6, spliceAI score ≥ 0.5.

^b^variant in: *CCT4*, score 0.94; *CDC27*, score 0.94; *HYDIN*, score 0.97; *LMNB2*, score 0.99; *PEX6*, score 0.96; *TNFSF14*, score 0.99.

^c^variant in *TCPR2*, score 0.99.

MAF, Minor Allele Frequency; AG, Acceptor Gain; DL, Donor Loss; het, heterozygous variant; na, not applicable.

Based on these observations and on segregation analyses by Sanger sequencing on the rest of the family, the NM_006729.4:c.868A>G (NP_006720.1:p.I290V) variant within exon 8 of the *DIAPH2* gene on chromosome X (Fig 1C, Table 1) was thus considered the most probable cause of NSHL in the family. The affected amino acid residue (isoleucine 290) is highly conserved and is located in the DID domain of the protein (Fig 1D).

The putative disease-causing variant co-segregates with the phenotype in the family, being present in the heterozygous state in female carriers (I2, II2, II4) and in the hemizygous state in the affected brothers (III1, III3). In addition, it is absent in the normal hearing brother (III2) and in one normal hearing male cousin (III5), who is the son of a carrier individual (Fig 1A). The identified variation is absent both in our in-house database of about 3,500 Italian exomes, and in a cohort of 125 Italian audiologically-tested normal-hearing controls. In addition, it is reported in the gnomAD database in the heterozygous state only in three European non-Finnish females (allele frequency: 1.816*10–5).

To look for further genetic evidence of the involvement of *DIAPH2* in NSHL, we screened for potentially pathogenic variants within exon 8 in 16 NSHL subjects, deriving from families compatible with a X-linked pattern of inheritance. No candidate pathogenic variations were found. In addition, the *DIAPH2* gene was submitted to GeneMatcher, a web-based tool designed to help the identification of additional individuals with variants in the same gene of interest [39]. A total of 7 subjects with hemizygous variants in the *DIAPH2* gene were identified, however, none of them matched with the HL phenotype [40]. At present, all these variations should be considered variants of unknown significance (VUS), as no experimental evidence in support of their pathogenicity, nor of their actual involvement in producing the associated phenotype, has been provided.

### The mouse ortholog of *DIAPH2* shows a specific spatio-temporal expression in the inner ear

**Fig 2:**
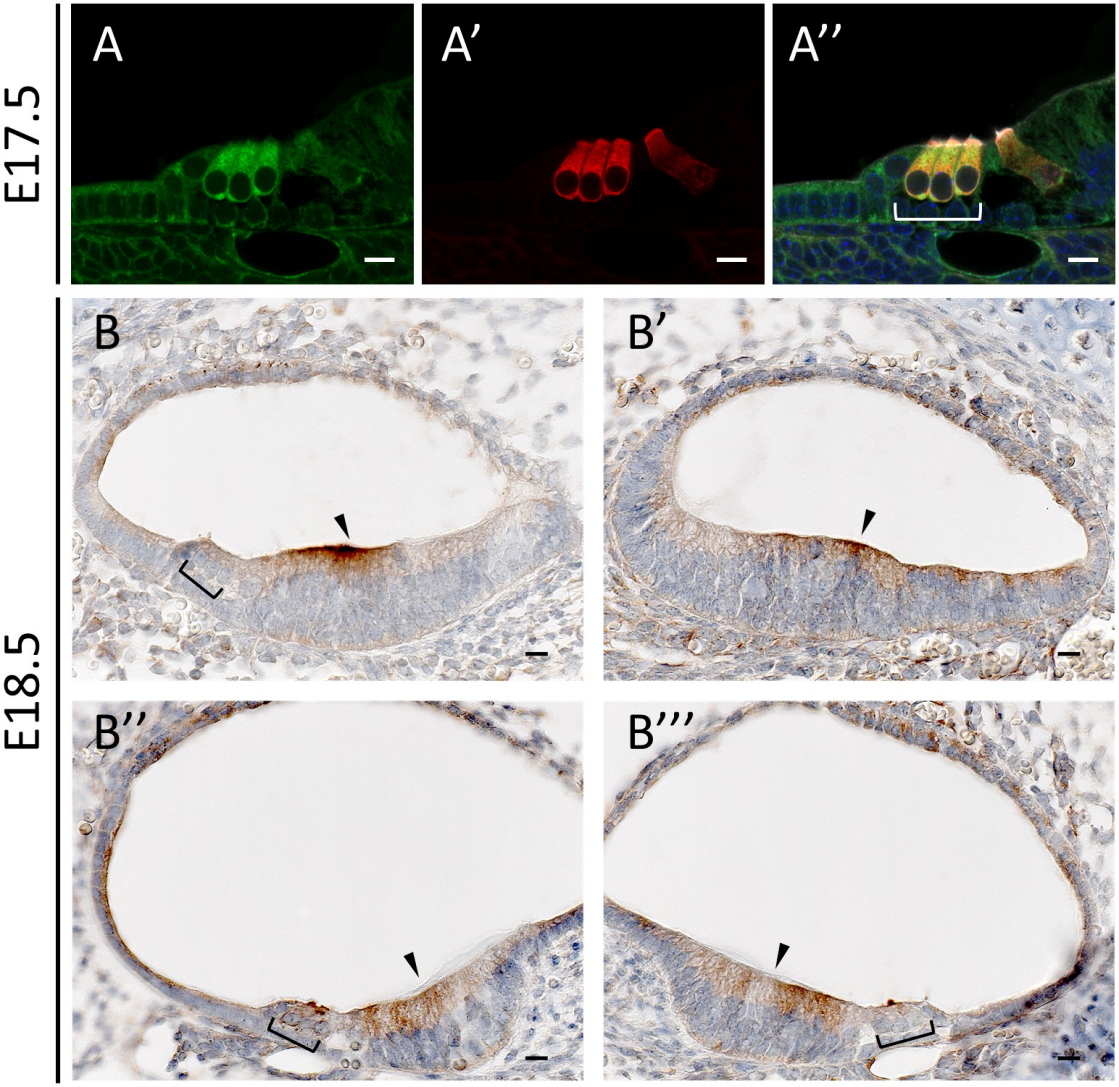
Diaph2 expression in E17.5 and E18.5 wild-type mouse cochlea. Brown indicates positive staining. (**A-A”**) Vibratome cross section of a E17.5 wild-type mouse cochlea showing Diaph2 in green (A) and the hair-cell marker Myosin VI in red (A’). The merged image of the red and green channels shows the co-localization of Diaph2 with the marker in OHCs. (**B-B”‘**) Cross section of the cochlear turns (B’: apical, B, B”‘: mid, B”: basal turn) of a E18.5 wild-type mouse fetus. At this stage, a different degree of cell differentiation can be observed from the base to the apex. The square brackets indicate OHCs and the arrowheads indicate Kölliker’s organ. Dorsal to the bottom. Scale bars 10 μm.

**Fig 3:**
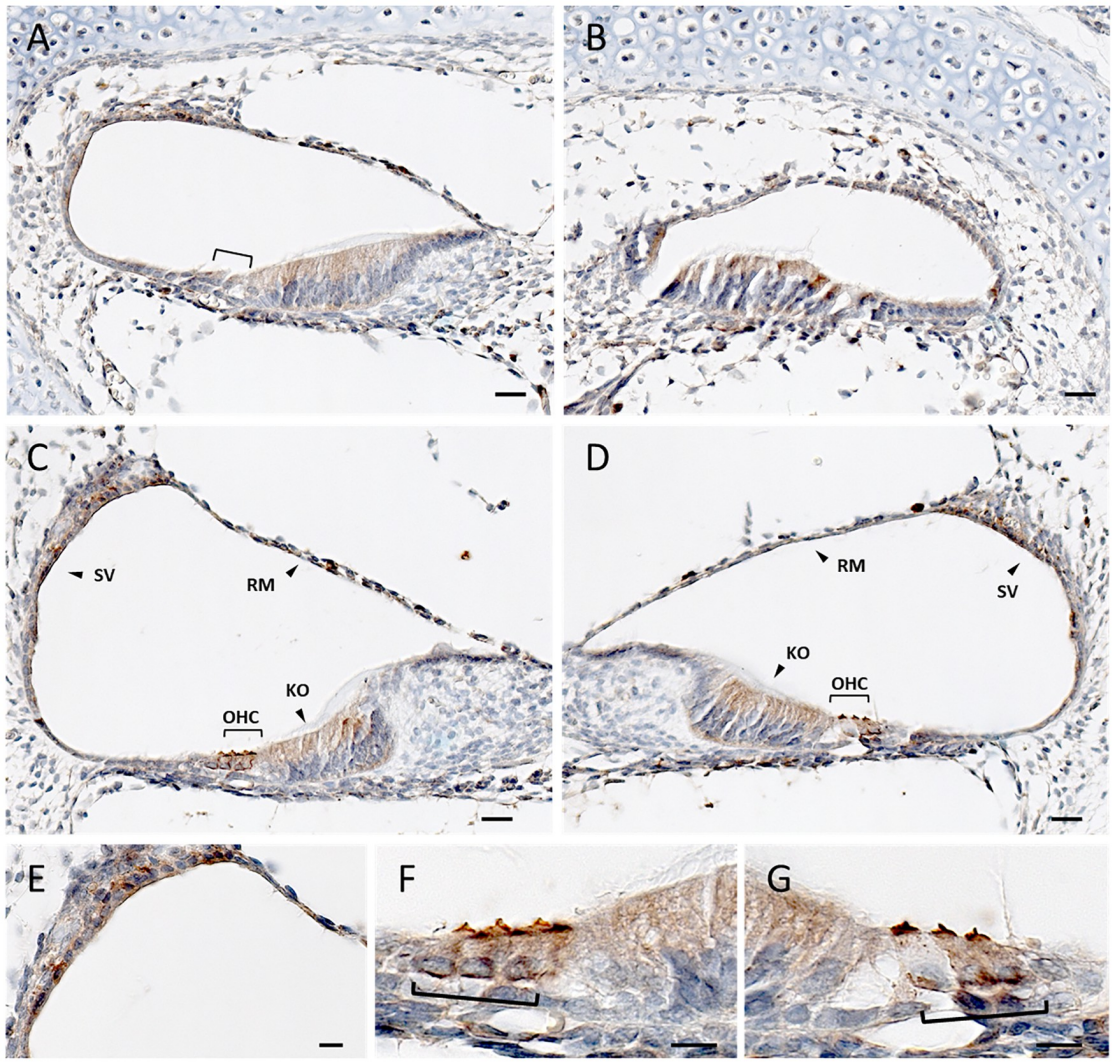
Diaph2 expression in P0 wild-type mouse cochlea. Brown indicates positive staining. (**A-D**) Cross section of the cochlear turns (B: apical, A, D: mid, C: basal turn) of a P0 wild-type mouse. At this stage, a different degree of cell differentiation can be observed from the base to the apex. The square brackets indicate OHCs (outer hair cells). The arrowheads indicate: Kölliker’s organ (KO), Reissner’s membrane (RM), stria vascularis (SV). Dorsal to the bottom. (**E**) Higher magnification of the stria vascularis in C. (**F-G**) Higher magnification of the OHC shown, respectively, in C and D. Scale bar: 10 μm.

**Fig 4:**
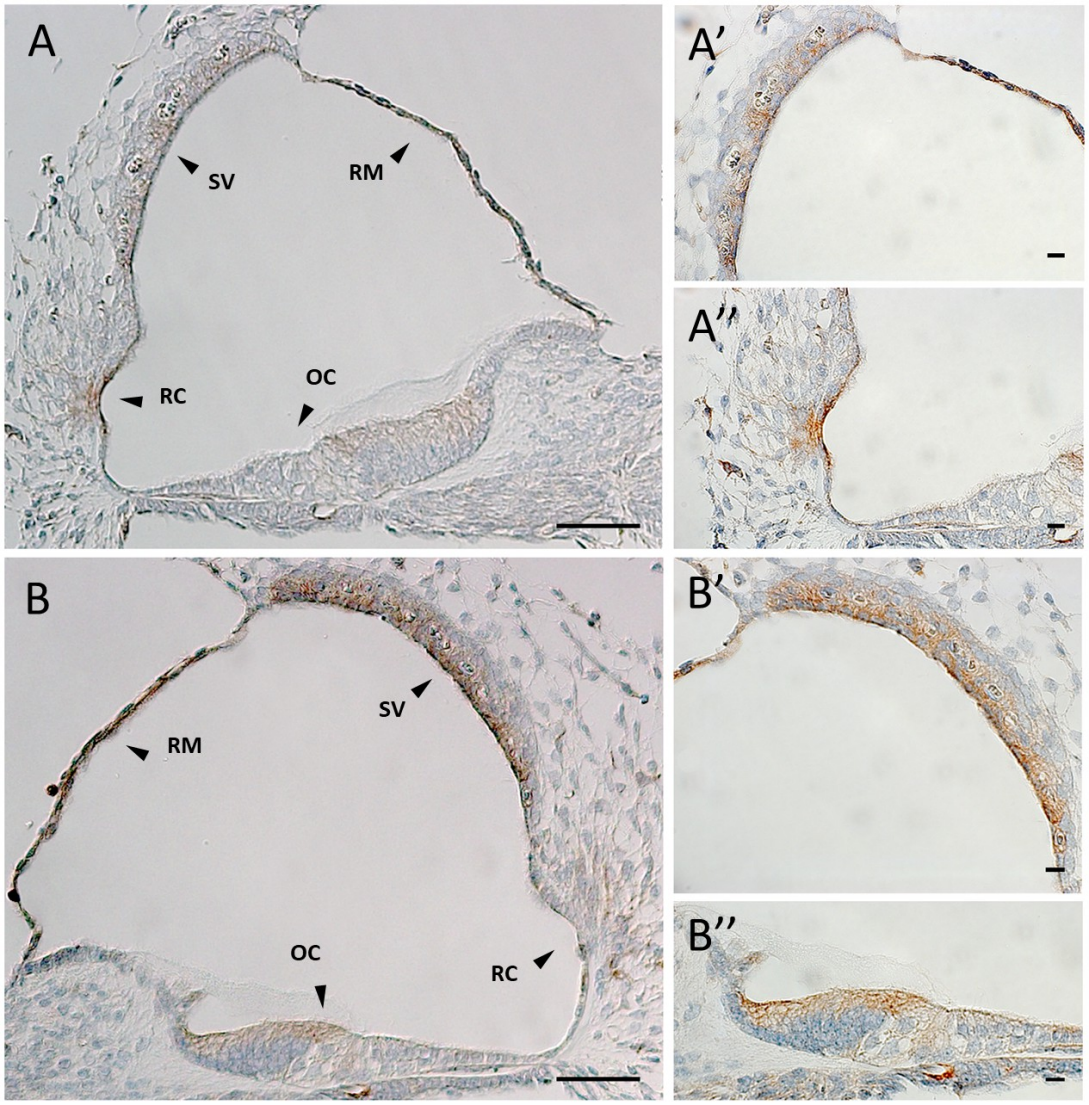
Diaph2 expression in P5 wild-type mouse cochlea. Brown indicates positive staining. (**A**) Scala media of the middle turn of cochlea. Scale bar 100 μm. (**A’-A”**) Higher magnification of the stria vascularis and of the outer sulcus shown in A. Scale bar 10 μm. (**B**) Scala media of the basal turn of cochlea. Scale bar 100 μm. (**B’-B”**) Higher magnification of the stria vascularis and of the organ of Corti shown in B. Scale bar 10 μm. Arrowheads point to: organ of Corti (OC), root cells (RC), Reissner’s membrane (RM), stria vascularis (SV).

To better understand the physiologic function of *DIAPH2* in hearing, we first verified the expression of *DIAPH2* mouse homolog (*Diaph2*) in the inner ear, by performing a reverse transcriptase-polymerase chain reaction (RT-PCR) assay on a sample of organ of Corti derived from a post-natal day 4 mouse (P4) [41]. A product of the expected size was amplified (S2 Fig in S1 File), suggesting that the gene, and likely the corresponding protein, are specifically expressed in the ear. Then, we performed immunohistochemical studies to evaluate the expression of the mouse ortholog protein Diaph2 in the mouse cochlea at different ages. Low Diaph2 expression was detected in the developing cochlear duct at embryonic days 14.5 and 16.5 (E14.5 and E16.5), with a similar localization of the protein in cells lining the cochlear duct. At this stage, Diaph2 is principally expressed in the dorsal wall of the cochlear duct, which will develop into the organ of Corti (S3 Fig in S1 File, n = 3 for each stage). In E18.5 (Fig 2) and P0 (Fig 3) wild-type mice, Diaph2 is highly expressed in the outer hair cells (OHCs), both in the stereocilia and at the base of OHCs (Fig 3F and 3G). The expression at the base of hair cells was detected also by immunostaining assays at P0 and P3 (S4 Fig in S1 File). In addition, it is expressed in Kölliker’s organ and in the stria vascularis. Interestingly, the expression varies along the developing cochlea in these stages. In fact, the epithelial cells of the cochlear duct start to differentiate from the base to the apex and Diaph2 is expressed in OHCs in the basal turn, while its expression towards the apex is localized in the luminal cells of the dorsal wall of the cochlear duct, similarly to the expression at earlier stages (Figs 2 and 3, n = 5 for each stage). The expression in OHCs was also confirmed by an immunofluorescence assay on a E17.5 mouse organ of Corti (Fig 2 and S1 File, containing Supplementary Methods). At P5, Diaph2 is expressed at low levels in the sensory epithelium, and it is mainly expressed in the stria vascularis, in the root cells and in the epithelial layer of Reissner’s membrane (Fig 4, n = 5). At older ages (P7 and P14 mice), Diaph2 was detected at very low levels in the stria vascularis only (S5 Fig in S1 File).

### The NM_006729.4:c.868A>G variant does not alter *DIAPH2* exon 8 splicing

As the c.868A>G variant in *DIAPH2* affects a nucleotide in exon 8 located only two base pairs upstream of the donor splice site, it was annotated not only as a missense, but also as a candidate splicing variant. *In-silico* predictions did not evidence any significant difference between the wild-type and mutant sequence in terms of the strength of the canonical donor splice site, however, the Human Splicing Finder tool [42] predicted the activation of a cryptic donor site, located just 2 nucleotides upstream of the canonical one, in the presence of the mutant c.868G allele (S6 Fig in S1 File). For this reason, we investigated the possible effect of the variant on *DIAPH2* splicing both *in vitro* and *in vivo*.

First, minigene transfection experiments–using the pBS-KS_modified hybrid minigene vector containing either wild-type or mutant exon 8 with the surrounding intronic sequences (S7A Fig and S1 File, containing Supplementary Methods)–were performed in three different human cell-lines (HeLa, HEK293, HepG2). Interestingly, in the very first experiment performed, the RT-PCR showed that the variant could induce exon 8 skipping. In addition, a different degree of exon 8 skipping was observed in the three cell lines analyzed, suggesting a tissue specificity of this splicing event (S7B Fig in S1 File). However, any attempt to replicate these results in the same cell lines failed, as no alternative splicing was observed in the cells transfected with the mutant construct in subsequent experiments (independent transfections, RNA extractions, and RT-PCR assays). Given the lack of reproducibility of the results, we performed the same minigene transfection experiments using a cell line more suitable for inner ear studies, i.e. the University of Bristol/Organ of Corti-2 (UB/OC-2) cell line, derived from the prosensory epithelium of the H-2Kb-tsA58 transgenic mouse [43, 44]. The RT-PCR analyses did not show any difference in the splicing pattern of UB/OC-2 cells transfected with the mutant minigene vector compared with those transfected with the wild-type, both in proliferative and differentiating conditions (S7B Fig in S1 File). The results were also confirmed by a competitive fluorescent RT-PCR assay, which showed a unique peak corresponding to the amplification of the transcript including the *DIAPH2* exon 8 (S7B Fig in S1 File). As an alternative approach, a 6.7 kb-long fragment spanning *DIAPH2* exons from 6 to 9 (including the entire 1.9 kb-long intron 8) was cloned into the pTARGET expression vector (S8A Fig in S1 File), and subjected to site-directed mutagenesis to obtain the mutant c.868A>G construct. The wild-type and mutant plasmids were independently and transiently transfected into HeLa, HEK293 and MDCK cell lines, and the presence of aberrantly spliced transcripts (e.g. derived from exon skipping, partial/complete intron retention, or activation of cryptic splice sites) was verified by RT-PCR and Sanger sequencing of the amplified products. No difference in *DIAPH2* splicing was detected in the presence of the variant compared to the wild-type (S8B Fig in S1 File).

Despite negative results from *in vitro* studies, we verified the possible consequences of the c.868A>G variant on *DIAPH2* splicing also *in vivo*, in both humans and mouse. *DIAPH2* transcript expression was evaluated on whole blood RNA from all available family members (II1, II2, III1, III2, III3), performing a RT-PCR analysis with exonic primers specifically designed to amplify exon 8 and its flanking exons. Only one amplification product with size compatible with that of the wild-type *DIAPH2* transcript was detected in all analyzed samples, independently from the genotype (S9A Fig in S1 File). Direct sequencing of the amplification products ruled out the presence of a 2-nt shorter exon 8 derived from the activation of a cryptic donor splice site, which would not be discriminated from the wild-type amplicon by agarose gel electrophoresis (S9B Fig in S1 File). In addition, we tested the possibility of haploinsufficiency due to the selective degradation of mutant *DIAPH2* transcripts by real-time RT-PCR. No significant difference in *DIAPH2* mRNA levels among genotypes was detected (S9C Fig in S1 File). We extracted RNA from the whole inner ears of *knock-in* mice carrying the corresponding nucleotide substitution (NM_172493.2:c.877A>G) found in the Italian family (see below). We then performed RT-PCR amplification and sequencing of exons 7–10 of *Diaph2*, and found no evidence of altered splicing in the mutant compared to the wild-type transcript (S10 Fig in S1 File).

### The NP_006720.1:p.I290V variant might alter RhoA-dependent activation of DIAPH2

**Fig 5:**
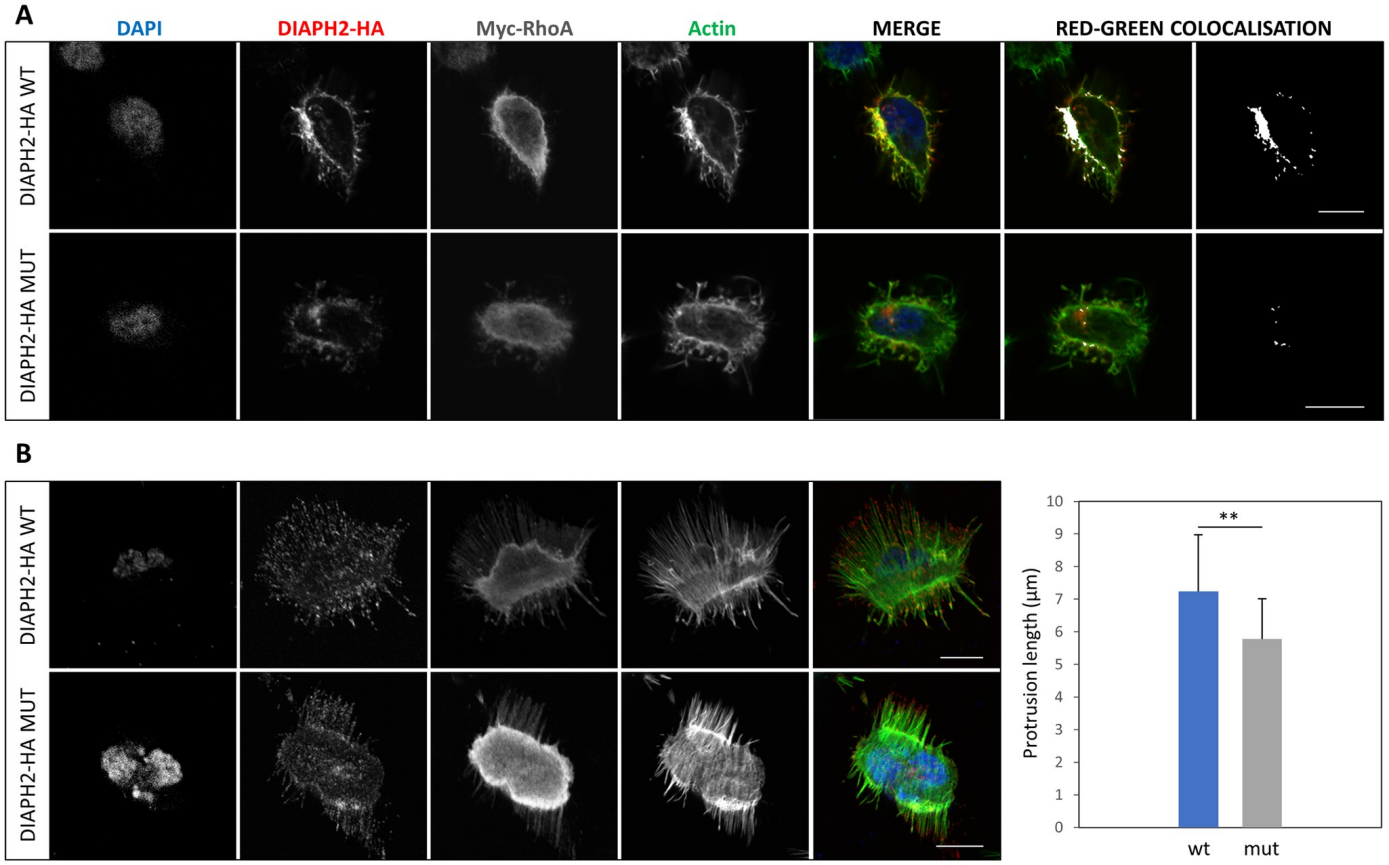
Evaluation of DIAPH2 localization and of the length of membrane protrusions in HEK293 under activating stimuli. DIAPH2 localization and cell morphology was evaluated in HEK293 cells 48 hours after transfection with plasmids coding for HA-tagged isoforms of wild-type or mutant DIAPH2 (DIAPH2-HA WT, DIAPH2-HA MUT), together with a Myc-tagged active form of RhoA (Myc-RhoA). (**A**) Single middle confocal sections for each channel and the merged image of the blue, red and green channels are shown. Image of colocalized pixels (in white, as generated by Fiji colocalization plugin) superimposed or not on a RG-merge are shown. (**B**) Single confocal sections of cells at the level of their adhesion to the glass are shown. The merged image of the blue, red and green channels is also shown. On the right, histograms representing the mean length of protrusions measured using Fiji in 20 wt and 20 mut HEK293 cells, with error bars showing the standard deviations. Significance level of unpaired t-test is shown (**: p<0.01). Images were acquired with Leica True Confocal Scanner (TCS) SP8 and are representative of 3 experiments. Scale bar: 10 μm.

The p.I290V variant is predicted as deleterious by 7 out of 8 different pathogenicity predictors (S11 Fig in S1 File). In addition, isoleucine 290 is highly conserved across vertebrates and lies in a region of the inhibitory domain possibly involved both in the auto-inhibition of the protein and in the interaction with Rho GTPases (Fig 1D). To evaluate whether the variation could affect DIAPH2 subcellular distribution, we performed immunofluorescence localization assays on HEK293 cells 48 hours after transfection with wild-type or mutant plasmids expressing an HA-tagged DIAPH2 protein isoform (pCMV-HA-hDIA2B_wt, pCMV-HA-hDIA2B_mut). These studies suggested that the variant does not alter DIAPH2 cytoplasmic localization, at least in basal conditions (S12 Fig in S1 File). As the p.I290V variant is located close to the region of interaction with RhoA, we analyzed the localization of wild-type and mutant DIAPH2 protein under activating stimuli, by performing the same immunofluorescence assay on HEK293 cells co-expressing wild-type or mutant DIAPH2 together with a constitutively active form of the activator RhoA (pCDNA3.1-Myc-RhoA-G14V). As expected, the activation of DIAPH2 by RhoA changed the localization of DIAPH2 from the cytoplasm to the plasma membrane. Co-localization with actin could be observed both for the wild-type and the mutant DIAPH2, however, the overlap of red and green signals was higher in cells expressing the wild-type protein (Fig 5A). In addition, the presence of RhoA promoted a morphological change of the cells, with the formation of actin-rich membrane protrusions in HEK293 cells (Fig 5B). Considering base sections of the cells (at the level of their adhesion to the glass), the DIAPH2 protein could be observed as red dots localized at the end of the actin bundles. Similar morphological changes were induced in cells expressing either the wild-type or the mutant DIAPH2 together with RhoA. Nevertheless, there was a statistically significant difference in the average length (measured with Fiji software [23]) of the membrane protrusions, which were ~20% shorter in cells expressing the mutant DIAPH2 (unpaired t test, p<0.01) (Fig 5B).

### Knock-out and knock-in *Diaph2* mouse mutants do not show an evident hearing defect

**Fig 6:**
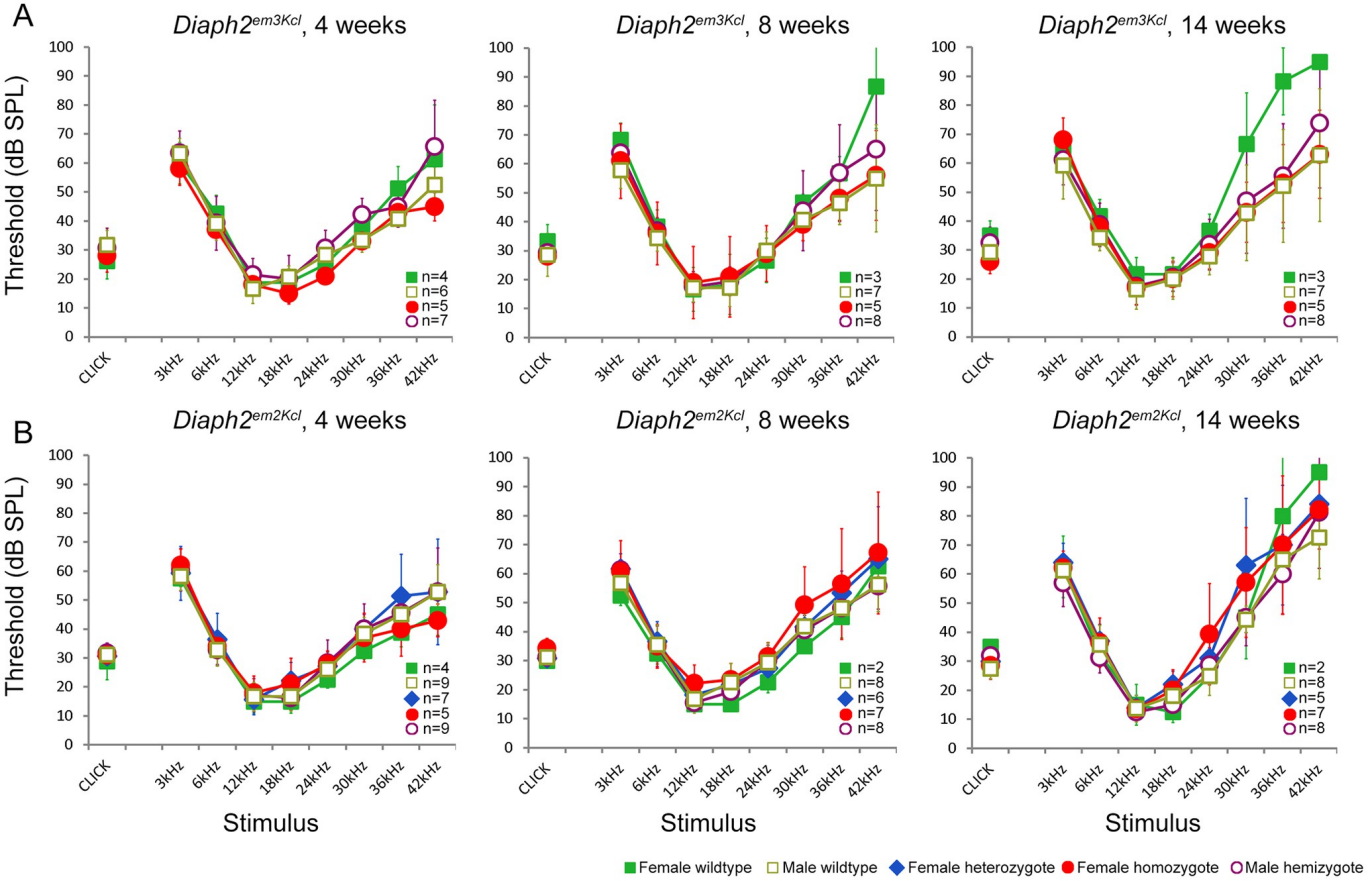
Analysis of auditory function in *Diaph2* mutant mice. (**A**) Mean ABR thresholds of mice carrying the knock-in variant (*Diaph2*^em3Kcl^) and (**B**) the knock-in variant plus a 19bp deletion (*Diaph2*^em2Kcl^) at 4 (left), 8 (center) and 14 (right) weeks of age. Male hemizygotes (empty maroon circles) and female homozygotes (filled red circles) have thresholds comparable to female heterozygotes (filled blue diamonds) and male and female wildtypes (squares, female filled green, male empty gold) at all ages. At 14 weeks old the *Diaph2*^em3Kcl^ female wildtypes have raised high-frequency thresholds compared to the other genotypes, but since there are only three of them and the seven male wildtypes have normal hearing, we suggest that this is not a relevant biological difference. Numbers of each genotype tested are shown on each plot. Error bars show standard deviation.

**Fig 7:**
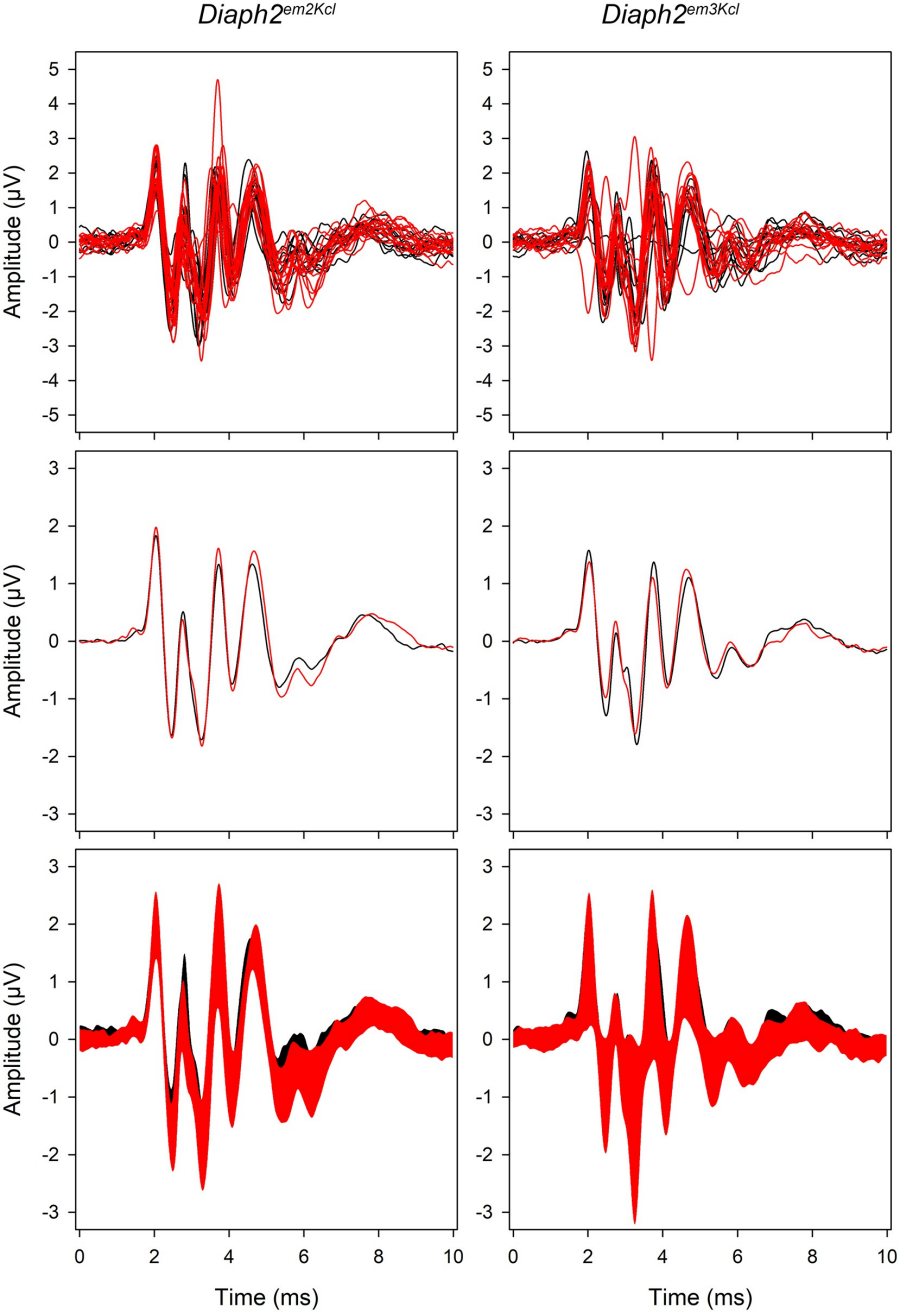
ABR waveform analysis in *Diaph2* mutant mice. ABR waveforms in response to a click stimulus shown at 30dB above threshold (sensation level, SL) at fourteen weeks old. The top panels show individual waveforms, the central panels show the mean waveform, and the bottom panels show the mean ± standard deviation. There is no obvious difference between *Diaph2*^em2Kcl^ hemizygote and homozygote mice (red, n = 15) and wildtype mice (black, n = 10) (left), or between *Diaph2*^em3Kcl^ hemizygous and homozygous mice (red, n = 13) and wildtype mice (black, n = 10) (right).

The auditory function of mice bearing the corresponding nucleotide substitution (NM_172493.2:c.877A>G, *Diaph2*^*em3Kcl*^) both as homozygous females and hemizygous males was evaluated at 4, 8 and 14 weeks of age by ABR recordings. ABR thresholds were not significantly different between mutant and wild-type genotypes at any of the time points (Fig 6). We also compared the waveforms of mutants with wild-type littermates to look for any indications of reduced amplitudes or prolonged latencies of waves, but there were no differences (Fig 7).

As we found no differences in auditory function between mice with the missense variant and controls, we also studied a second mutant line that carried the 877A>G missense substitution but also a 19bp deletion nearby created by CRISPR off-target effects (*Diaph2*^*em2Kcl*^), predicted to result in a frameshift and create a premature stop codon in exon 9. No impairment of ABR thresholds or waveforms was observed at the ages tested: 4, 8 and 14 weeks old (Figs 6B and 7). These results indicate that neither the missense NM_172493.2:c.877A>G(NP_766081.1:p.I293V) variant in *Diaph2* nor the disruption of the transcript affects the development of normal hearing in mice.

## Discussion

In the last decade, exome sequencing has proved a powerful tool for detecting potentially pathogenic variants underlying NSHL, allowing both the screening of genes already known to be associated with deafness and the discovery of new disease-causing genes. However, the actual demonstration of the causal role of potentially pathogenic variants—especially when found in novel candidate genes—still represents a critical and limiting step. Here, exome variant prioritization, including specific analysis of synonymous variants and CNVs, pointed to the NM_006729.4:c.868A>G (p.I290V) variant in *DIAPH2* as the most likely candidate for prelingual HL in an Italian NSHL family, which was supported by segregation analyses. Interestingly, the here reported variant in *DIAPH2* was hypothesized to result in a loss-of-function effect. Indeed, the hearing phenotype is inherited as a likely recessive trait in the family, and heterozygous carriers of the variant have normal hearing. This is in contrast with the mechanism of action of mutations in the other two diaphanous genes, *DIAPH1* and *DIAPH3*, which lead either to a gain-of-function effect or to the overexpression of the protein, respectively, and are transmitted as dominant alleles [4, 10, 12]. Despite the efforts to find a second NSHL family with potentially pathogenic variants in *DIAPH2*, we still lack additional genetic data confirming the association of this gene with deafness. On one hand, this is not surprising, as there are other examples of genes with a clear causal association with deafness that have been reported to be mutated in just one family, such as *CLRN2* [45]. On the other hand, we cannot exclude the presence of intronic/intergenic variants or other complex rearrangements that are not detectable using exome sequencing, in this or other genes.

Unlike the other two members of the diaphanous-related formin family, *DIAPH1* and *DIAPH3*, little is known about the physiologic role of *DIAPH2* in hearing. Our immunohistochemistry studies in wild-type mice now give some insights into the expression profile of the Diaph2 protein in the inner ear. Indeed, Diaph2 showed a low level of labelling in the developing cochlear duct at E14.5 and E16.5, and a specific strong expression in outer hair-cell stereocilia at later stages (E17.5, E18.5 and P0). By P5, Diaph2 expression was mainly restricted to structures important for the cochlear fluid homeostasis (e.g. root cells, stria vascularis). These observations point to a possible involvement of Diaph2 in the development of stereocilia of OHCs, supporting a role for DIAPH2 as an actin regulator in the cochlear sensory cells. Moreover, the expression of Diaph2 –postnatally—in structures required for fluid homeostasis and endocochlear potential maintenance, suggest other possible roles of the protein in hearing function.

Encouraged by the specific expression pattern of Diaph2 in the cochlea and the reduced length of protrusions in cells transfected with mutant *Diaph2*, we produced *Diaph2* mutant mice carrying the corresponding variant identified in the here described NSHL family by CRISPR/Cas9 technology, and characterized their auditory phenotype. We also produced mice carrying a 19bp deletion predicted to result in a truncated transcript. ABR recordings, performed in 4-, 8-, and 14-week-old mice, did not indicate any differences in ABR thresholds or waveforms in mice carrying either variant as compared to wild-type littermates. Both mutants also showed completely normal startle responses using a calibrated click as a stimulus. The phenotype of a knock-out model of *Diaph2* was recently reported by the International Mouse Phenotyping Consortium (IMPC, *Diaph2*^*tm1b(EUCOMM)Hmgu*^ allele, http://www.mousephenotype.org/data/genes/MGI:1858500, last accessed on 27 May 2022). These mice have exons 6 and 7 of *Diaph2* deleted and show ABR thresholds in the normal range (not significantly different from controls).

Although the lack of hearing phenotype in mutant mice does not help in supporting our hypothesis of a causal relationship between *Diaph2* and deafness, there are few examples of genes causing hearing loss in humans that do not cause the same phenotype in mice. For instance, *SLC22A4* was identified in 2016 as responsible for ARSNHL in two unrelated consanguineous families of Tunisian ancestry, and later on in a Moroccan family and in an additional Tunisian pedigree [46–48]. The *SLC22A4* knockout mouse, made available by the International Mouse Phenotyping Consortium, shows no apparent signs of hearing loss, with evoked auditory brain stem responses not significantly different between wild-type mice and homozygous mutants (https://www.mousephenotype.org/data/genes/MGI:1353479). There are also examples of gene causing a hearing defect in mouse but not in humans. For instance, *HCN1* knockout mice display a hearing phenotype, including abnormal auditory brainstem responses, impaired acoustic startle reflex, reduced gap detection and spatial localization [49] (https://www.mousephenotype.org/data/genes/MGI:1096392). However, there are no reports to date of *HCN1* loss-of-function variants in humans associated with deafness. On the contrary, gain-of-function variants in the same gene cause an epileptic encephalopathy in humans [50].

In our case, the absence of a hearing phenotype in *Diaph2* mutant mice may be due to functional redundancy in the *Diaph* gene family, with Diaph1 and/or Diaph3 compensating for the lack of Diaph2. In support of this hypothesis, it was reported that the *Diaph1* knock-out mice did not recapitulate the microcephaly phenotype described in humans with the *DIAPH1* homozygous truncating mutation [51]. Conversely, *Diaph1*/*Diaph2* double knock-outs showed several abnormalities in neural development [52]. Indeed, while both Diaph1 and Diaph2 proteins are required for the assembly of the apical actin belt in neuroepithelial cells, the loss of Diaph1 only does not affect this structure [51]. A similar redundancy of *Diaph* genes might thus also exist in the cochlea.

Concerning the actual pathogenic effect of the NM_006729.4:c.868A>G (p.I290V) variant, immunofluorescence studies of DIAPH2 wild-type and mutant proteins did not demonstrate a major effect of the p.I290V missense variant on DIAPH2 protein localization. Instead, a statistically significant -although modest- reduction in the average length of the RhoA-induced membrane protrusions in HEK293 cells expressing mutant DIAPH2 was observed. As actin filaments are essential components of the sensory hair-cell stereocilia, whose length is precisely regulated [53], it is possible that even a modest disruption of these finely regulated structures could have an effect on the signal transmission.

In conclusion, we show that *DIAPH2* is expressed in the developing inner ear and might be involved in regulating actin dynamics in cochlear sensory cells. We propose that a hemizygous missense variant in *DIAPH2* (p.I290V) on the X-chromosome may underlie the progressive hearing impairment in a family of Italian origin, possibly impairing RhoA-dependent activation of DIAPH2. Further screening of the *DIAPH2* gene in other families and different populations will provide important information about the frequency of NSHL-associated variants in this gene. Additional molecular studies will also be needed to better clarify the role of *DIAPH2* in normal hearing and deafness.

## Supporting information

S1 File This file containing all the supplementary material (tables, figures and methods) mentioned in the manuscript is available. (ZIP)
