# Supplementary material for: In-depth genetic and molecular characterization of diaphanous related formin 2 (DIAPH2) and its role in the inner ear
Source: PLoS One. 2023 Jan 23;18(1):e0273586. doi: 10.1371/journal.pone.0273586 (PMC9870134; doi:10.1371/journal.pone.0273586)
Supplement: S1 File — (ZIP) [file pone.0273586.s001.zip › SupplementaryInformation/Figure_S9.pdf]

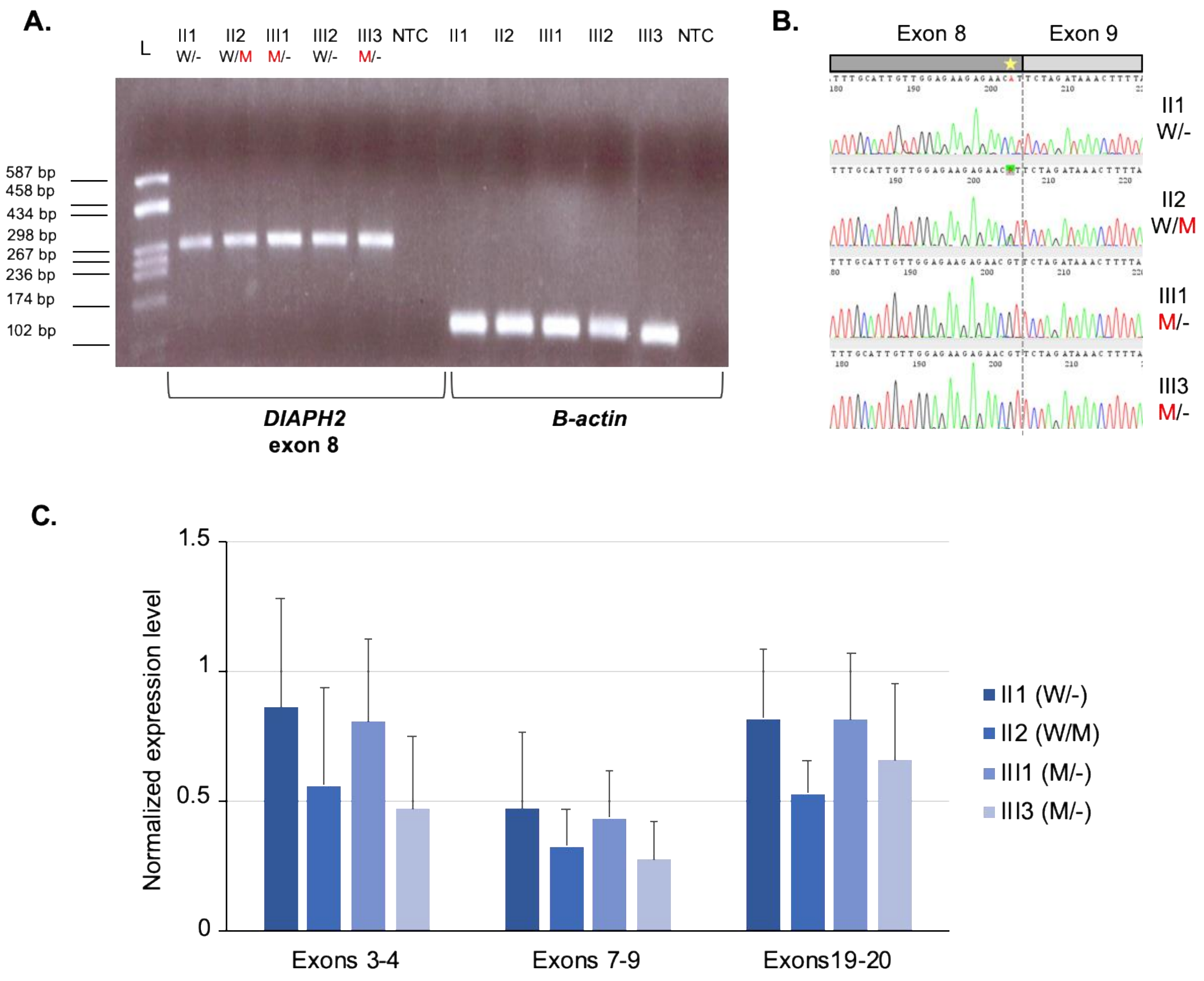

Figure S9. Analysis of *DIAPH2* exon 8 splicing in blood from NSHL3 family subjects. A. An RT-PCR assay was performed on cDNA obtained from whole blood of all available family members, using primers located in exon 6 and 9, respectively (301 bp). As positive control, the β-actin (ACTB) housekeeping gene was amplified. An aliquot (10 microliters) of each RT-PCR reaction was loaded into a 2% agarose gel. Only the product corresponding to the amplification of a wild-type transcript is visible in all samples. L: DNA ladder, used as molecular weight marker (pUC9/*HaeIII*); NTC: no template control. The genotype of each individual is indicated, as follows W: wild-type allele, M: mutant allele, - hemizygous (Y chromosome). **B.** Electropherograms showing the nucleotide sequences of RT-PCR amplification products, confirming that the amplified fragment correspond to the wild-type DIAPH2 transcript. The junction between exon8 and 9 is indicated by a dashed gray line. The location of the c.868A>G variation is marked with a yellow star. The genotype of each individual is indicated, as follows W: wild-type allele, M: mutant allele, - hemizygous. **C.** Real-time RT-PCR showing *DIAPH2* mRNA expression in NSHL3 family subjects (the genotype of each individual is indicated, as follows W: wild-type allele, M: mutant allele, - hemizygous). Each assay was repeated twice (on different input cDNA) and performed in triplicate (technical replicate). Data are presented as normalized expression levels calculated by the GeNorm software, using as housekeeping genes both *HMBS* and *GAPDH*. Error bars represent the standard deviation of the geometric mean.
