# Supplementary material for: In-depth genetic and molecular characterization of diaphanous related formin 2 (DIAPH2) and its role in the inner ear
Source: PLoS One. 2023 Jan 23;18(1):e0273586. doi: 10.1371/journal.pone.0273586 (PMC9870134; doi:10.1371/journal.pone.0273586)
Supplement: S1 File — (ZIP) [file pone.0273586.s001.zip › SupplementaryInformation/Figure_S8.pdf]

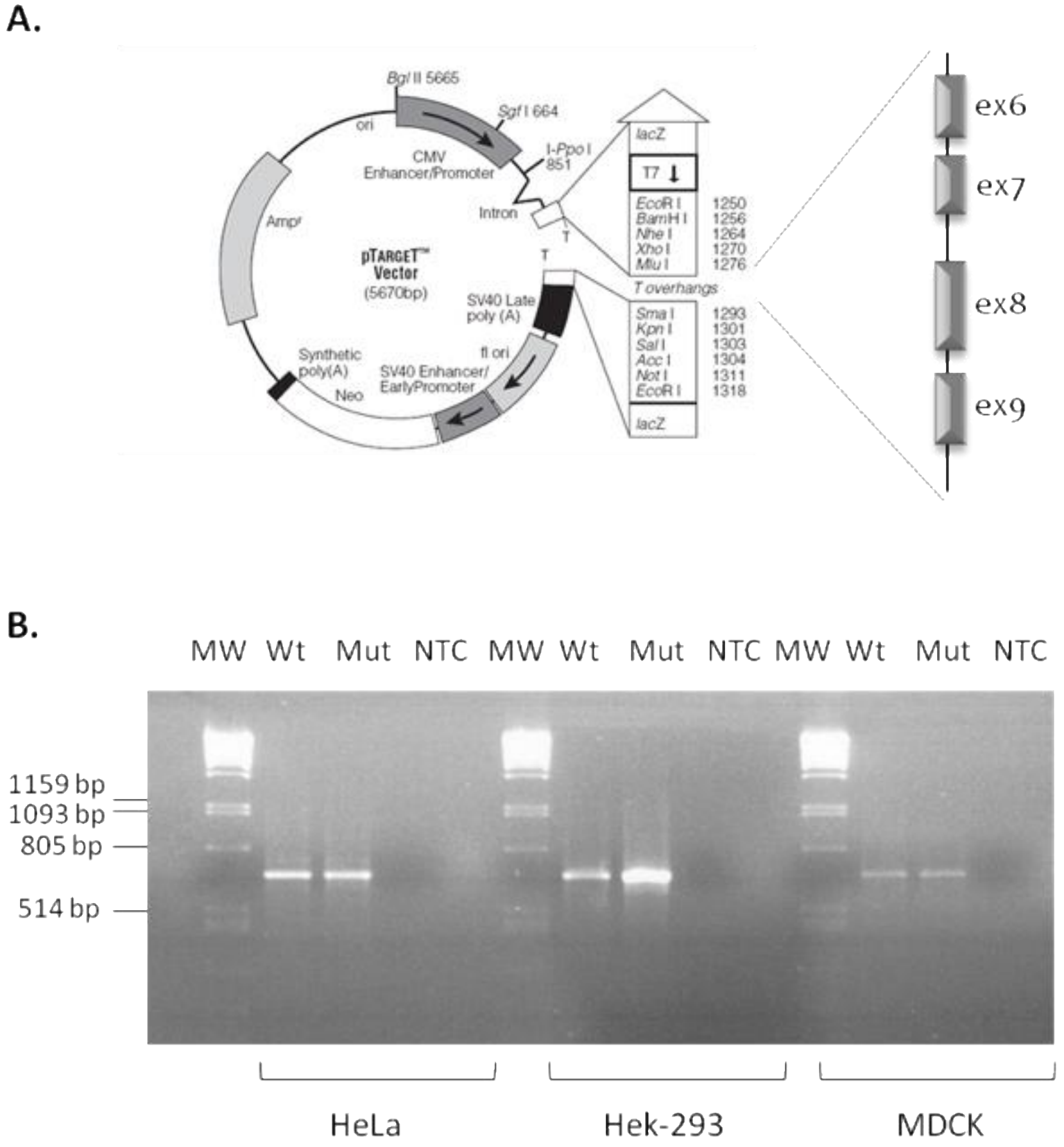

Figure S8. In vitro characterization of the effect of the c.868A>G variation on splicing using a *DIAPH2* minigene spanning exons 6 to 9. A. Schematic representation of the pTARGET mammalian expression vector (Promega) and the portion of the *DIAPH2* gene cloned (exons 6, 7, 8 and 9). A fragment of about 6,700 bp was PCR amplified from the genomic DNA of a *DIAPH2* wild-type individual and then subjected to site-directed mutagenesis in order to obtain the mutant version. **B**. In all experiments, 1 or 2 µg of each recombinant plasmid were transiently transfected in 3 different human cell lines (HeLa, HeK-293, and MDCK,Madin-Darby canine kidney cells). RNA extraction was performed 24 hours after transfections, and RT-PCR analysis was carried out using a forward primer complementary to the pTARGET T7 promoter sequence and a reverse oligonucleotide designed on DIAPH2 exon 9. The agarose gel of RT-PCRs is shown. MW: molecular weight marker; Wt: cells transfected with the vector containing the wild-type nucleotide (A); Mut: cells transfected with the vector containing the mutation (G); NTC: no template control. The obtained ~660-bp-long band corresponds to the inclusion of exon 8 in the mature transcript, as confirmed by Sanger sequencing analysis.
