# Supplementary material for: In-depth genetic and molecular characterization of diaphanous related formin 2 (DIAPH2) and its role in the inner ear
Source: PLoS One. 2023 Jan 23;18(1):e0273586. doi: 10.1371/journal.pone.0273586 (PMC9870134; doi:10.1371/journal.pone.0273586)
Supplement: S1 File — (ZIP) [file pone.0273586.s001.zip › SupplementaryInformation/Figure_S12.pdf]

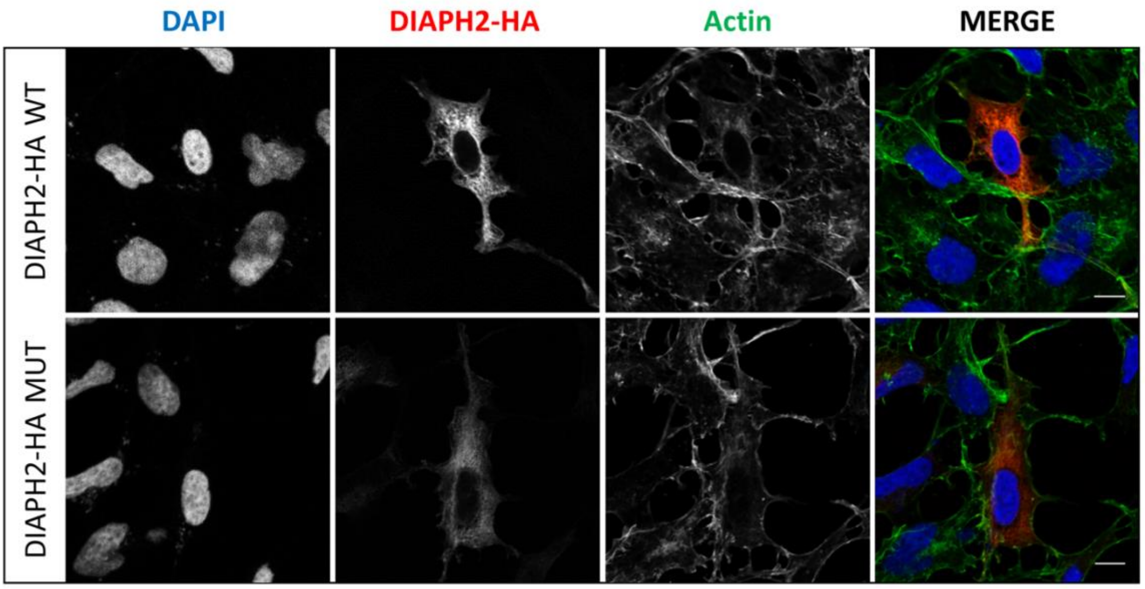

Figure S12. DIAPH2 immunolocalization studies in basal conditions. DIAPH2 localization was studied in HEK293 cells 48 hours after transfection with plasmids coding for HA-tagged isoforms of wild-type or mutant DIAPH2 proteins (DIAPH2-HA-WT, DIAPH2-HA-MUT). Images were acquired with Leica True Confocal Scanner (TCS) SP8. Single confocal sections are shown. DAPI: 4',6-diamidino-2-phenylindole. Scale bar: 10 μm.
