# Supplementary material for: In-depth genetic and molecular characterization of diaphanous related formin 2 (DIAPH2) and its role in the inner ear
Source: PLoS One. 2023 Jan 23;18(1):e0273586. doi: 10.1371/journal.pone.0273586 (PMC9870134; doi:10.1371/journal.pone.0273586)
Supplement: S1 File — (ZIP) [file pone.0273586.s001.zip › SupplementaryInformation/Figure_S10.pdf]

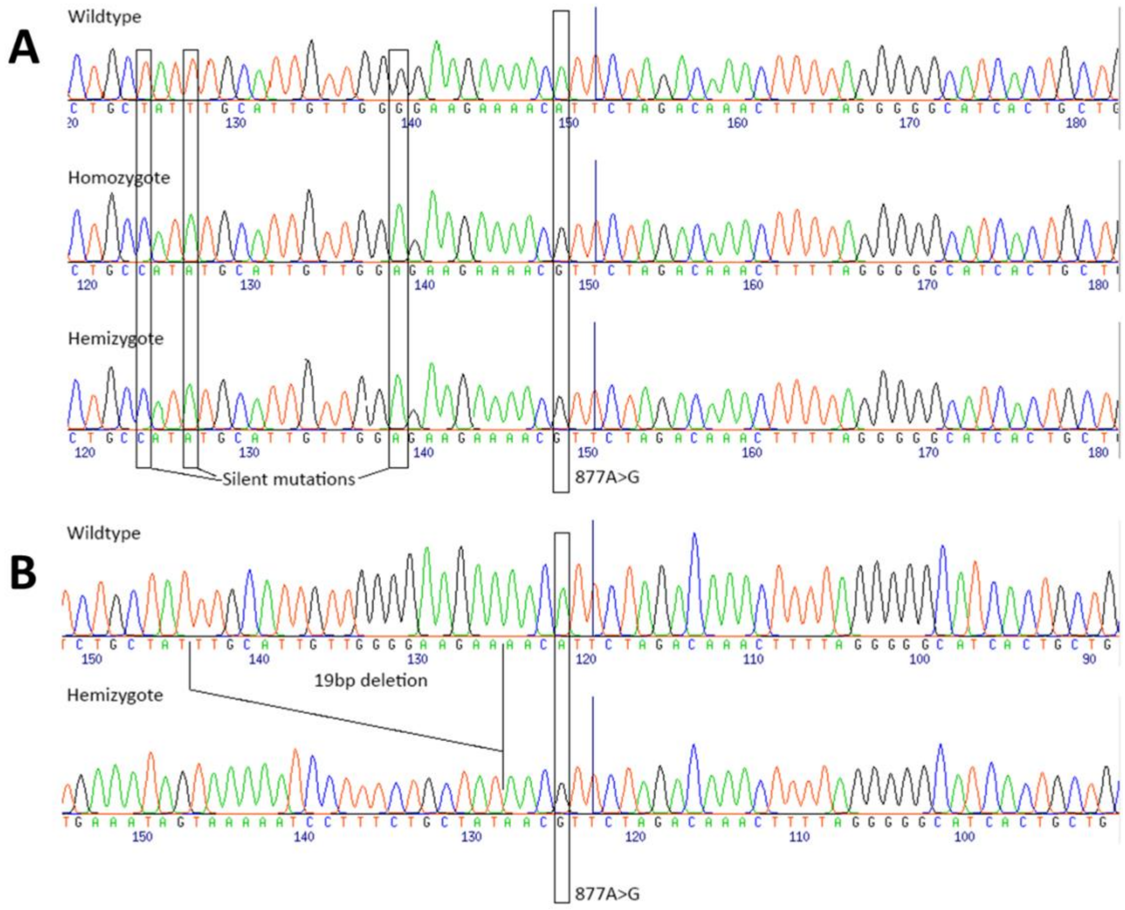

Figure S10. ***In-vivo*** **analysis of** ***Diaph2*** **exon 8 splicing in the mouse cochlea.** Traces of *Diaph2* cDNA from mice carrying the 877A>G variant knocked in (*Diaph2*^{em3Kcl}), along with either some silent mutations (**A**) or with a 19bp deletion just preceding 877G (*Diaph2*^{em2Kcl}) (**B**). The vertical line indicates the first base of exon 8. No alternative splicing is visible in either mutant.
