# Supplementary material for: In-depth genetic and molecular characterization of diaphanous related formin 2 (DIAPH2) and its role in the inner ear
Source: PLoS One. 2023 Jan 23;18(1):e0273586. doi: 10.1371/journal.pone.0273586 (PMC9870134; doi:10.1371/journal.pone.0273586)
Supplement: S1 File — (ZIP) [file pone.0273586.s001.zip › SupplementaryInformation/Figure_S11.pdf]

	DIAPH2 variant
**Predictor**	**p.I290V**
**SIFT**	Tolerated
**Polyphen 2 HumDiv**	Possibly Damaging
**Polyphen 2 HumVar**	Possibly Damaging
**MutationTaster**	Disease causing
**Mutation Assessor**	FI M
**FATHMM**	Damaging
**Condel**	Deleterious
**CADD**	23.4

Figure S11. Pathogenicity prediction of the p.I290V missense variant with 8 commonly used software. FI, Functional Impact of a variant; CADD, Combined Annotation Dependent Depletion. FI is ranked as: high (H), medium (M) for predicted functional variants and low (L) for predicted non-functional variants. CADD Scaled C-score represents the PHRED-like [-10*log10(rank/total)] score, ranking a variant relative to all possible substitutions of the human genome (8.6x109).
