# Supplementary material for: In-depth genetic and molecular characterization of diaphanous related formin 2 (DIAPH2) and its role in the inner ear
Source: PLoS One. 2023 Jan 23;18(1):e0273586. doi: 10.1371/journal.pone.0273586 (PMC9870134; doi:10.1371/journal.pone.0273586)
Supplement: S1 File — (ZIP) [file pone.0273586.s001.zip › SupplementaryInformation/File_S1_Methods.pdf]

Supplementary Methods

In vitro splicing analyses

For *in-vitro* splicing assays on *DIAPH2* exon 8, the genomic DNA region containing the NM_006729.4:c.868A>G variant was PCR amplified from the genomic DNA of a healthy individual (with primers including *NdeI* restriction sites: 5'-ggaattccatatgCAAGGAGCAACAACTCCAAGA-3' and 5'-ggaattccatatgTCTGCTCAATCACTTTGAATCC-3') and cloned in the hybrid alpha-globin-fibronectin minigene plasmid (modified pBS-KS) [Baralle et. Al., 2003]. The obtained plasmid was subjected to site-directed mutagenesis in order to generate the mutant version. HeLa, HEK293 and HepG2 cell lines were cultured according to standard procedures. UB/OC-2 cells, deriving from the organ of Corti of E13 temperature-sensitive Immortomouse [Rivolta and Holley, 2002], were cultured in Minimum Essential Medium (MEM; Thermo Fisher Scientific, Waltham, MA, USA) supplemented with GlutaMAX, 10% FBS and 50 Units/ml γ-IFN (PeproTech, Cranbury, NJ, USA) and grown at 33°C in a humidified atmosphere of 5% CO_2, in order to maintain cells in the proliferative state. To differentiate the cells, γ-IFN was rinsed off and cells were cultured in MEM with only GlutaMAX and 10% FBS at 33°C for 2 days and then moved to 39°C in a humidified atmosphere of 5% CO_2. For splicing studies, an equal number of cells ($2.5*10^5$ HeLa or UB/OC-2 in proliferative state; $3*10^5$ HEK293, HepG2 or UB/OC-2 in differentiating conditions) were transiently transfected with 1 μg of either the wild-type or the mutant recombinant pBS-KS vector. Cells transfected with the empty pBS-KS vector were used as negative control (mock). The Jet-PRIME reagent (Euroclone, Wetherby, UK) was used for transfections in HeLa, HEK293 and HepG2 cells, whereas Lipofectamine 2000 (Thermo Fisher scientific, Waltham, MA, USA) was used as transfection reagent in the UB/OC-2 cell line. Total RNA was isolated from cells 24 hours after transfection, using the EuroGold TriFast reagent (Euroclone). Random hexamers and the ImProm-II Reverse Transcriptase (Promega, Madison, WI, USA) were used to perform first-strand cDNA synthesis, starting from 500 ng of total RNA, according to the manufacturer's instructions. Of a total of 20 μL of the RT reaction, 1 μL was used as template for amplifications, using primers annealing to the flanking α-globin/FN1 exonic sequences (α2-3: 5'-CAACTTCAAGCTCCTAAGCCACTGC-3' and Bra2: 5'-CAATGGATGGGGGTGGAG-3').

In vivo splicing analysis (human)

RT-PCRs were performed using 1 μL of cDNA derived from patients' whole-blood RNA and the following primers: 5'- GCTGGAAAAGCTTCTGGACA-3' and 5'-TGGTGAAAATCGTTCCCTGT-3'. RT-PCRs were performed under standard conditions using the GoTaq DNA Polymerase (Promega) on a Mastercycler EPgradient (Eppendorf, Hamburg, Germany), using extension times up to 5 minutes, to detect the retention of intron8 (1.9 kb), if present. Direct sequencing of amplified products was performed on both strands using the BigDye Terminator Cycle Sequencing Ready Reaction Kit v.1.1 and an automated ABI-3500 DNA sequencer (Thermo Fisher Scientific, Waltham, MA, USA).

Real-time RT-PCR were also performed to measure *DIAPH2* expression levels. Primers for three different amplicons were designed (exon 3-4: forward 5'-TCAAGTCGCGATGAGTGAGT-3', reverse 5'-CCATCTCACGTTTGGTGGTA-3'; exon7-9: forward 5'-CTTATTCAATGCCTCAAAGC-3', reverse 5'-GGTGAAAATCGTTCCCTGTT-3'; exons 19-20: forward 5'-TCGCATGCCATATGAAGACA-3', reverse 5'-GTTCAGGCTCACAGAGGTCA-3'). All reactions were performed with the 2X SYBR Premix Ex Taq II (Takara Bio Inc, Otsu, Japan) in a final volume of 20 μL, following standard cycling conditions on a Light Cycler 480. Each assay was repeated twice (on different input cDNA) and performed in triplicate (technical replicate). Data were analyzed

with GeNorm [Vandensompele et al., 2002], using as housekeeping genes for normalization both hydroxymethylbilane synthase (HMBS) and Glyceraldehyde 3-phosphate dehydrogenase (GAPDH).

In vivo splicing analysis (mouse)

The inner ears of adult mice were dissected out and stored at -20°C in RNAlater stabilization reagent (Ambion). RNA was extracted using QIAshredder columns (QIAgen, cat. no. 79654) and the RNeasy mini kit (QIAgen, cat. no. 74104), following the manufacturer's instructions. RNA concentration was measured using a Nanodrop spectrophotometer (ND-8000). RNA was normalized to the same concentration between littermates, then treated with DNAse 1 (Sigma, cat. no: AMPD1) before cDNA was made using Precision Reverse Transcription Premix (PrimerDesign, cat. no: RT-premix2). RT-PCR was carried out using primers designed against exon 7 (Diaph2_x7F: TCAGTACAAAGTCATTCAGTGCC) and exon 10 (Diaph2_x10R: CATATGGAGAGGTGACAAGTGC). The PCR product was cleaned using Illustra ExoProStar (Cytiva, cat no: US78210) and Sanger sequenced by Source BioSciences (Nottingham, UK).

Immunofluorescence colocalization studies on vibratome cochlear sections

Dissected cochleas from E17.5 mouse fetuses were fixed in 4% paraformaldehyde for 30 minutes and embedded in 5% agarose. Cochleas were sectioned at 60 µm thick using a VF-300-0Z CompressTome Vibrating Microtome (Precisionary Instruments, Greenville, NC, USA), permeabilized with 2% TritonX-100 in PBS and blocked in blocking solution (10% FBS, 0.5% TritonX-100 in PBS). The sections were incubated with primary antibodies (1:50: anti-Dia2 antibody, sc-55540, Santa Cruz Biotechnology; 1:500: anti-MyoVI antibody, 25-6791, Proteus BioSciences, Ramona, CA, USA) overnight and, subsequently, with secondary antibodies (Alexa Fluor 488 anti-mouse, A21202; Alexa Fluor 555 anti-rabbit, A31572, both 1:500 diluted, Invitrogen, Carlsbad, CA, USA) in blocking solution and mounted in glycerol using Imaging Spacers (Grace Bio-Labs, Bend, OR, USA). Images were acquired with Zeiss LSM 880 Airyscan microscope (Zeiss, Oberkochen, Germany).

Supplementary References

Zwaenepoel I.; Mustapha M.; Leibovici M.; Verpy E.; Goodyear R.; Liu X. Z.; Nouaille S.; Nance W. E.; Kanaan M.; Avraham K. B.; et al. Otoancorin, an inner ear protein restricted to the interface between the apical surface of sensory epithelia and their overlying acellular gels, is defective in autosomal recessive deafness DFNB22. Proc Natl Acad Sci USA, 2002,99, 6240-6245, doi: 10.1073/pnas.082515999.

Vona, B.; Hofrichter, M. A.; Neuner, C.; Schr√∂der, J.; Gehrig, A.; Hennermann, J. B.; Kraus, F.; Shehata-Dieler, W.; Klopocki, E.; Nanda, I.; et al. DFNB16 is a frequent cause of congenital hearing impairment: implementation of STRC mutation analysis in routine diagnostics. Clin Genet, 2015, 87, 49-55, doi:10.1111/cge.12332.

Baralle, M.; Baralle, D.; De Conti, L.; Mattocks, C.; Whittaker, J.; Knezevich, A.; Ffrench-Constant, C.; Baralle, F. Identification of a mutation that perturbs NF1 gene splicing using genomic DNA samples and a minigene assay. J Med Genet 2003, 40, 220–222, doi:10.1136/jmg.40.3.220.

Rivolta M.N.; Holley M.C. Cell lines in inner ear research. J Neurobiol 2002, 53, 306-318.

Vandesompele, J.; De Preter, K.; Pattyn, F.; Poppe, B.; Van Roy, N.; De Paepe, A. Speleman, F. Accurate normalization of real-time quantitative RT-PCR data by geometric averaging of multiple internal control genes. Genome Biol 2002, 3, RESEARCH0034. doi: 10.1186/gb-2002-3-7-research0034.
