# Supplementary material for: In-depth genetic and molecular characterization of diaphanous related formin 2 (DIAPH2) and its role in the inner ear
Source: PLoS One. 2023 Jan 23;18(1):e0273586. doi: 10.1371/journal.pone.0273586 (PMC9870134; doi:10.1371/journal.pone.0273586)
Supplement: S1 File — (ZIP) [file pone.0273586.s001.zip › SupplementaryInformation/Figure_S3.pdf]

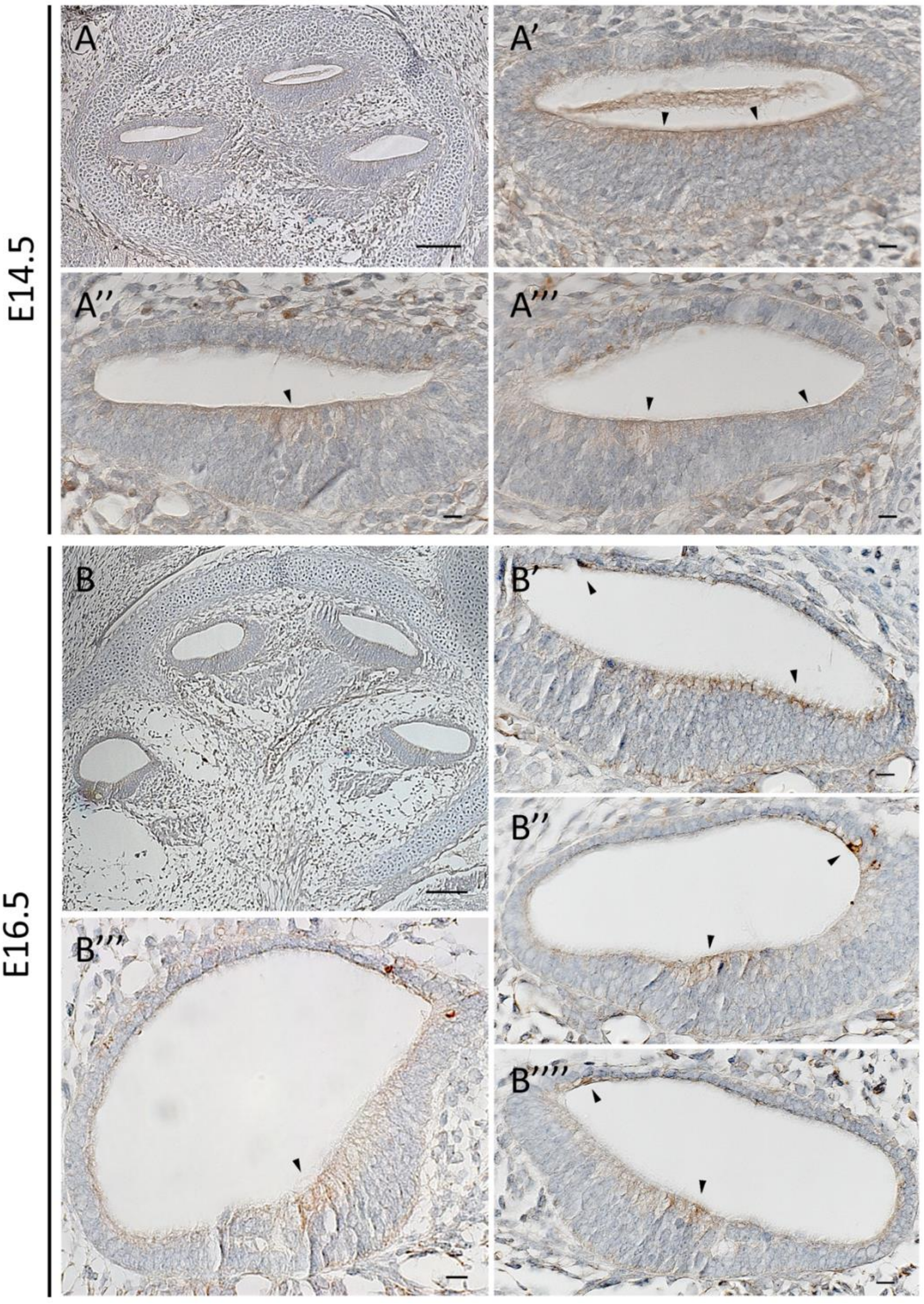

Figure S3. Diaph2 expression in E14.5 and E16.5 wild-type mouse cochlea. A. Cross section of the cochlea of a E14.5 wild-type mouse embryo. At this stage, the scala vestibuli and scala tympani have not yet developed, and only one chamber – the developing scala media – is present. Also, the different cells composing the organ of Corti have not differentiated yet. Dorsal to the bottom. Scale bar: 100 µm. **A’-A’’’.** Higher magnification of the turns of the developing cochlear duct. Diaph2 expression is indicated by arrowheads. Scale bars 10 µm. **B.** Cross section of the cochlea of a E16.5 wild-type mouse fetus. At this stage, the scala vestibuli and scala tympani begin to open starting from the base of the cochlea. Dorsal to the bottom. Scale bar: 100 µm. **B’-B’’’.** Higher magnification of the apical (B’), middle (B’’, B’’’’) and basal (B’’’) turns of the developing cochlear duct. Arrowheads point to Diaph2 expression. Scale bars 10 µm.
