# Supplementary material for: In-depth genetic and molecular characterization of diaphanous related formin 2 (DIAPH2) and its role in the inner ear
Source: PLoS One. 2023 Jan 23;18(1):e0273586. doi: 10.1371/journal.pone.0273586 (PMC9870134; doi:10.1371/journal.pone.0273586)
Supplement: S1 File — (ZIP) [file pone.0273586.s001.zip › SupplementaryInformation/Figure_S2.pdf]

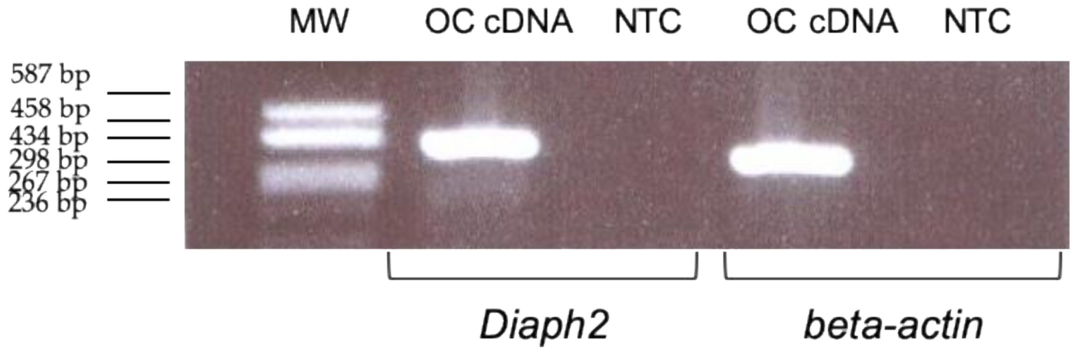

Figure S2. Expression of mouse Diaph2 mRNA in P4 organ of Corti by RT-PCR. Agarose gel showing the amplification of *Diaph2* by RT-PCR, using primers located in exon 6 and 10, respectively. As positive control, the beta-actin (Actb) housekeeping gene was amplified. In both cases, RNA was extracted from the organ of Corti of a P4 mouse. In brief, the tissue was firstly mechanically disrupted in the presence of 700 µl of QIAzol Lysis Reagent (QIAGEN) and then homogenized using a QIAshredder homogenizer (QIAGEN). After a short (2 min) centrifugation at 13000 rpm, a classical phenol- chloroform extraction was performed. The obtained RNA was quantified, reverse transcribed to cDNA with random nonamers and the SuperScriptIII Reverse Transcriptase enzyme (Invitrogen), and finally PCR amplified. OC: organ of Corti; MW: molecular weight marker; NTC: no template control.
