# Supplementary material for: In-depth genetic and molecular characterization of diaphanous related formin 2 (DIAPH2) and its role in the inner ear
Source: PLoS One. 2023 Jan 23;18(1):e0273586. doi: 10.1371/journal.pone.0273586 (PMC9870134; doi:10.1371/journal.pone.0273586)
Supplement: S1 File — (ZIP) [file pone.0273586.s001.zip › SupplementaryInformation/Figure_S1.pdf]

A. Click-evoked potential ABR

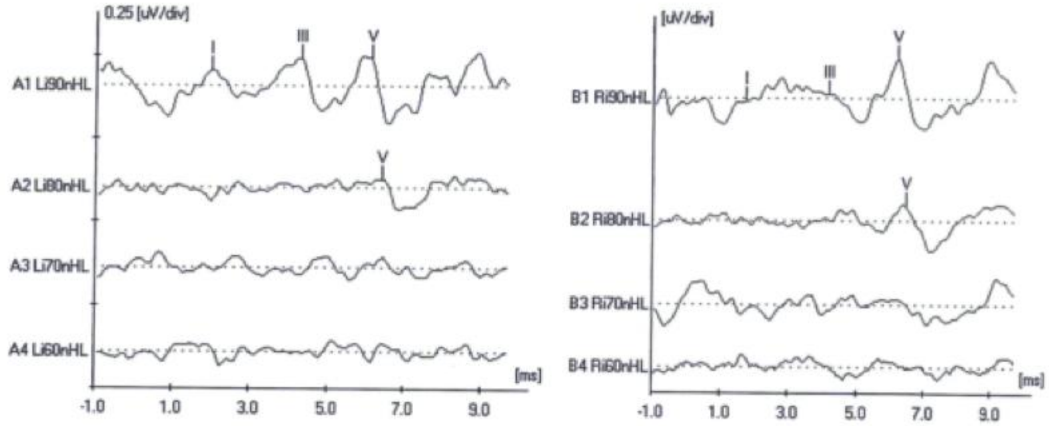

B. Tone burst ABR

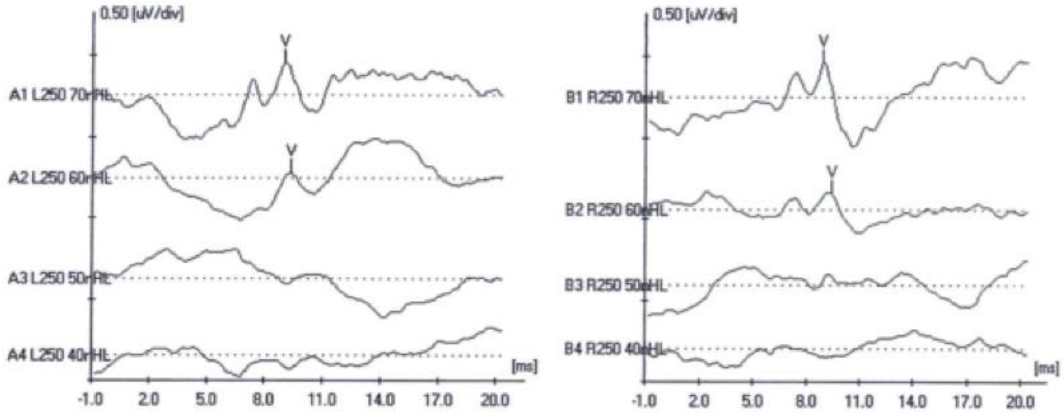

Figure S1. Analysis of auditory brainstem evoked potentials in proband III3. (**A**) Click-evoked potential ABR. The ABR were performed when the subject was aged 4. For both ears, waves I, III, and V were present at the starting intensity of 90 dB nHL. Wave V was replicable at 80 dB nHL but disappeared at 70 dB nHL. (**B**) Toneburst ABR. A 450-500 Hz toneburst was performed using a Blackman ramp and rarefaction polarity.
