# Supplementary material for: In-depth genetic and molecular characterization of diaphanous related formin 2 (DIAPH2) and its role in the inner ear
Source: PLoS One. 2023 Jan 23;18(1):e0273586. doi: 10.1371/journal.pone.0273586 (PMC9870134; doi:10.1371/journal.pone.0273586)
Supplement: S1 File — (ZIP) [file pone.0273586.s001.zip › SupplementaryInformation/Figure_S5.pdf]

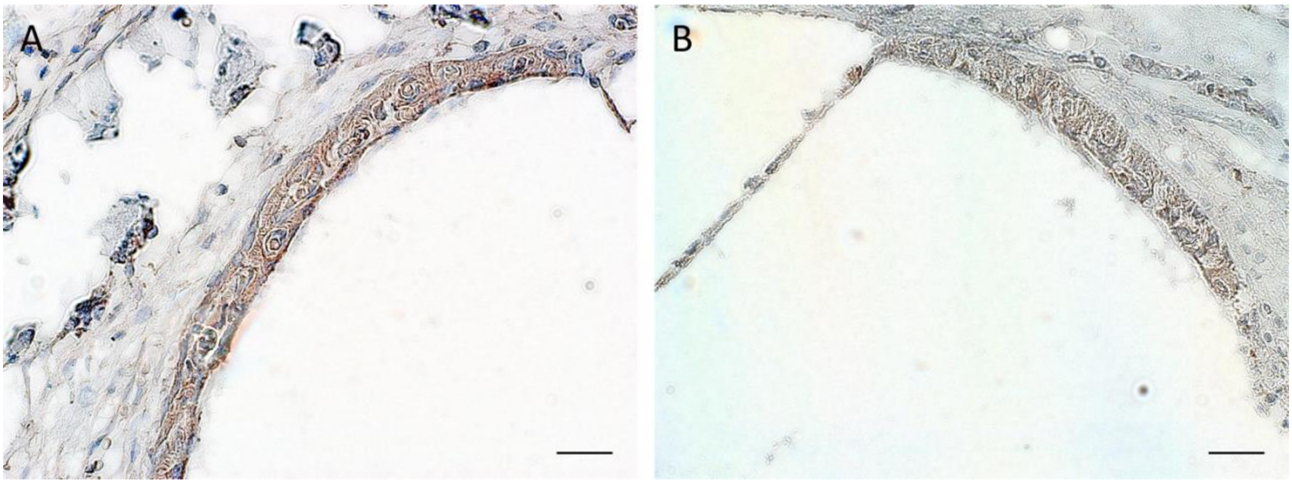

Figure S5. Diaph2 expression in P7 and P14 wild-type mouse cochlea. Magnification of the cochlear basal turn Cross section of the cochlea of a P7 (A) and P14 (B) wild-type mouse showing the stria vascularis. Brown indicates Diaph2 staining. Scale bar: 10 µm.
