# Supplementary material for: In-depth genetic and molecular characterization of diaphanous related formin 2 (DIAPH2) and its role in the inner ear
Source: PLoS One. 2023 Jan 23;18(1):e0273586. doi: 10.1371/journal.pone.0273586 (PMC9870134; doi:10.1371/journal.pone.0273586)
Supplement: S1 File — (ZIP) [file pone.0273586.s001.zip › SupplementaryInformation/Figure_S4.pdf]

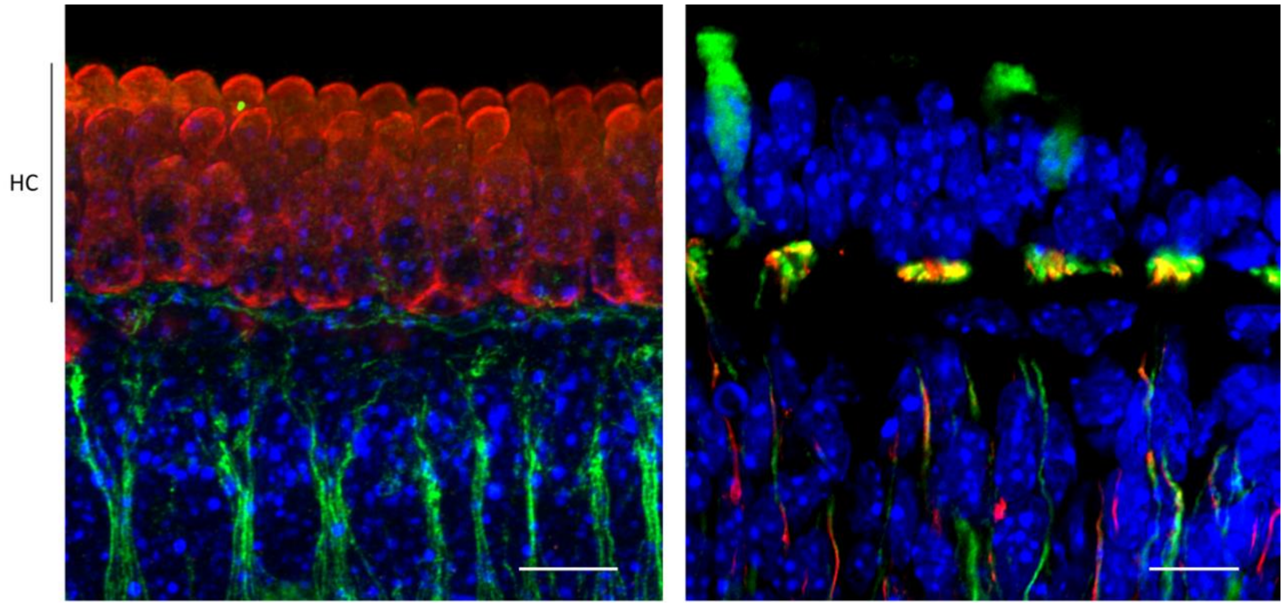

Figure S4. Evaluation of Diaph2 expression in whole-mount mouse cochleas. Immunofluorescence on whole mount cochlear dissections from P0 (left, maximum intensity projection) and P3 (right, single confocal section) mice. Diaph2 is shown in green, whereas red indicates the hair-cell marker Myosin VIIa (left) or the neuron marker Tuj1 (right). Nuclei are shown in blue (DAPI). HC: hair cells. Scale bar: 10 µm. In brief, dissected cochleas from wild-type mice were fixed in 4% paraformaldehyde, permeabilized in 0.1% Triton X-100 and blocked in blocking solution (10% FBS in PBS). Cochleas were incubated overnight with primary antibodies: mouse anti-Dia2 (1:50, sc-55540, Santa Cruz Biotechnology) and rabbit anti-MyoVIIa (left, 1:500, 25-6790, Proteus BioSciences Ramona, CA, USA) or rabbit anti-Tuj1 (right, 1:1000, T2200, Sigma-Aldrich). Finally, cochleas were incubated with secondary antibodies (Alexa Fluor 488 anti-mouse, A21202; Alexa Fluor 555 anti-rabbit, A31572, both 1:500 diluted, Invitrogen, Carlsbad, CA, USA) in blocking solution and counterstained with DAPI. Images were acquired with Zeiss LSM 880 Airyscan microscope (Zeiss, Oberkochen, Germany).
