# Supplementary material for: In-depth genetic and molecular characterization of diaphanous related formin 2 (DIAPH2) and its role in the inner ear
Source: PLoS One. 2023 Jan 23;18(1):e0273586. doi: 10.1371/journal.pone.0273586 (PMC9870134; doi:10.1371/journal.pone.0273586)
Supplement: S1 File — (ZIP) [file pone.0273586.s001.zip › SupplementaryInformation/Figure_S6.pdf]

A.

Prediction algorithm	Donor splice site (hg19 coordinates)	Score (strenght)		Variation
		DIAPH2 exon8 wild-type (c.868A)	*DIAPH2* exon8 mutant (c.868G)	
**SpliceView**	Canonical (chrX:96171574)	88 (medium)	84 (medium)	4.5%
**NetGene2**	Canonical (chrX:96171574)	0.82 (medium)	0.83 (medium)	1.2%
**Human Splicing Finder**	Canonical (chrX:96171574)	89 (medium)	89 (medium)	0%
	Cryptic (chrX:96171572)	39.29 (below threshold)	66.43 (weak)	69.08%
**Splice Site Prediction by Neural Network**	Canonical (chrX:96171574)	0.99 (strong)	0.99 (strong)	0%

B.

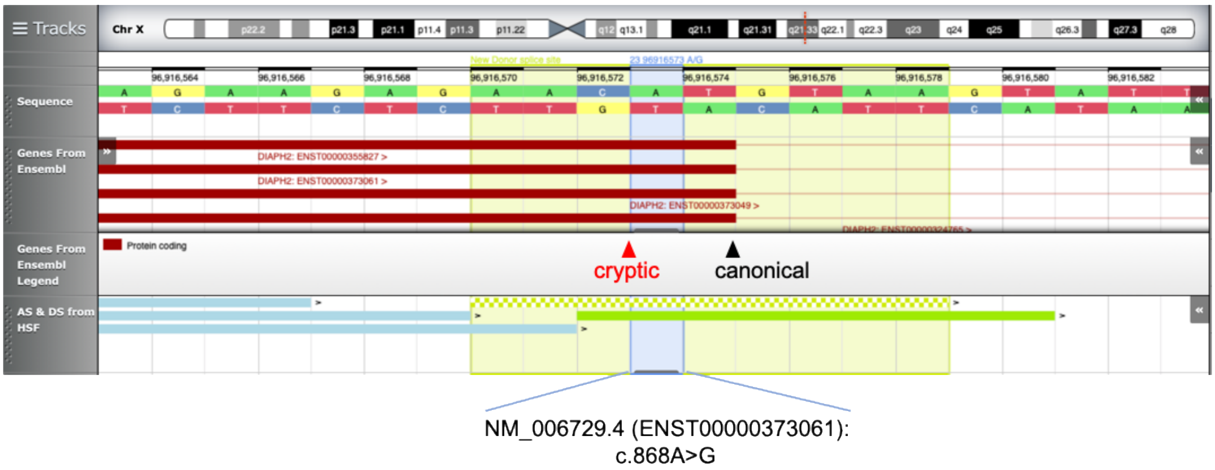

Figure S6. *In-silico* analysis of the impact of c.868A>G variant on *DIAPH2* pre-mRNA splicing. A. Table reporting the prediction of donor splice site strength for wild-type and mutant DIAPH2 exon8 sequence. Predictions were performed using several different tools: SpliceView (http://bioinfo.itb.cnr.it/~webgene/wwwspliceview.html, last accessed 18 November 2020), NetGene2 (http://www.cbs.dtu.dk/services/NetGene2/, last accessed 18 November 2020), Human Splicing Finder (https://hsf.genomnis.com/, last accessed 19 November 2020), Splice Site prediction by neural networks (https://www.fruitfly.org/seq_tools/splice.html, last accessed 18 November 2020).
Splice sites with a value of 60-80 (0.6-0.8) correspond to weak sites; 80-90 (0.8-0.9) to medium strength sites; >90 (0.9) to strong sites. Increase or decrease in site score ≥10% are considered to significantly impact on splicing. **B**. Screenshot of the Human Splicing Finder prediction results on *DIAPH2* c.868A>G (RefSeq reference sequence: NM_006729.4, Ensembl reference sequence ENST00000373061). The mutant position is highlighted in blue, with the reference sequence (GRCh38-hg38 genome annotation) displayed below the X chromosome ideogram. A black and a red arrow indicate the canonical and the cryptic donor splice site, respectively.
