# Supplementary material for: In-depth genetic and molecular characterization of diaphanous related formin 2 (DIAPH2) and its role in the inner ear
Source: PLoS One. 2023 Jan 23;18(1):e0273586. doi: 10.1371/journal.pone.0273586 (PMC9870134; doi:10.1371/journal.pone.0273586)
Supplement: S1 File — (ZIP) [file pone.0273586.s001.zip › SupplementaryInformation/Figure_S7.pdf]

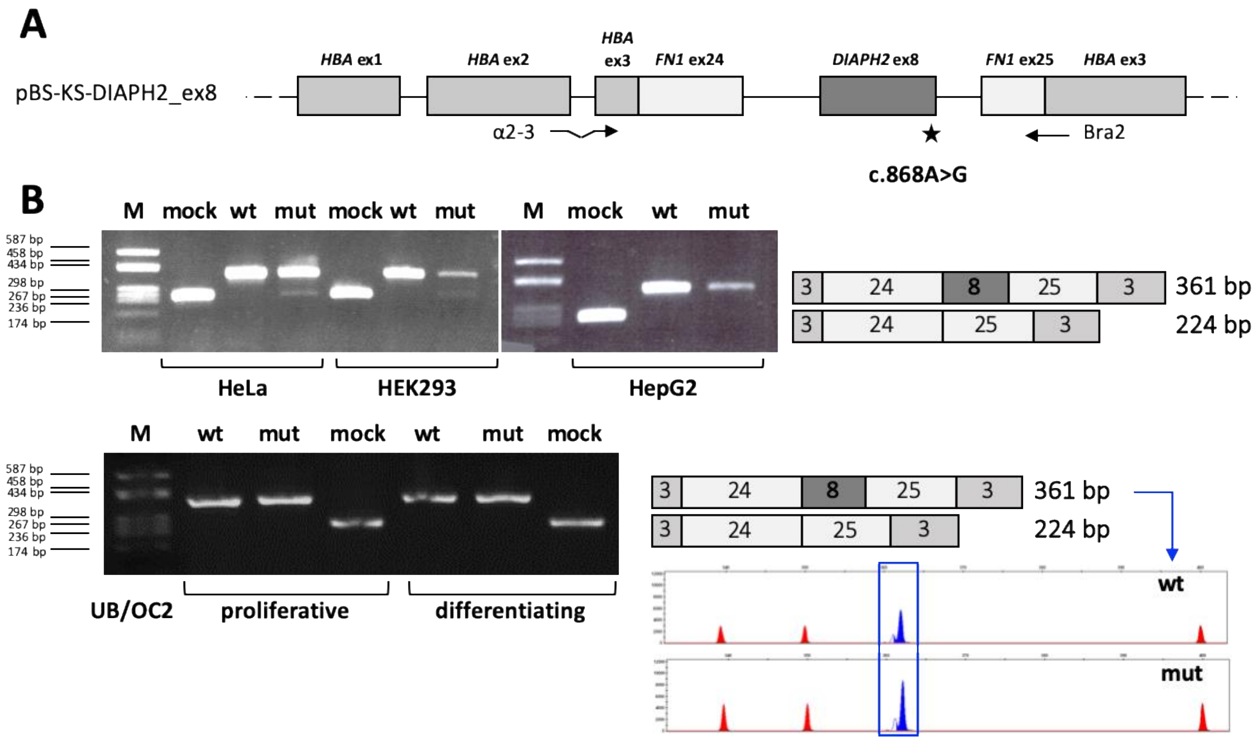


**Figure S7. *In-vitro* analysis of the impact of c.868A>G variant on *DIAPH2* pre-mRNA splicing.** **A.** Schematic representation of the hybrid pBS-KS-*DIAPH2*_ex8 minigene where α-globin (*HBA*) exons are represented by light grey boxes, fibronectin (*FN1*) exons by white boxes, whereas introns are shown as black lines (not to scale). Exon 8 of *DIAPH2* is represented by a dark grey box. The c.868A>G variant in exon 8 is indicated by a black star. Black arrows represent the primers used in RT-PCR assays. **B.** On the left, agarose gel electrophoresis of RT-PCR products obtained from RNA of HeLa, HEK293, HepG2 cells (upper panel) and UB/OC-2 cells under proliferative or differentiating conditions (lower panel), transfected with the wild-type (wt), mutant (mut), or empty (mock) minigene vector. M: molecular weight marker (pUC9/*HaeIII*). On the right, schematic representation of the splicing products, as verified by Sanger sequencing. The length of each fragment is shown. The presence of a single amplification product corresponding to the full-length *DIAPH2* transcript was also verified by fluorescent RT-PCR, using a FAM-labelled forward primer. An aliquot of the RT-PCR was run on an automated ABI-3500 DNA sequencer, together with an internal size standard (ROX-500 HD, Thermo Fisher Scientifics), and the results analyzed with the Peak Scanner v.1.0 software. A Peak Scanner window displaying the fluorescence peak (boxed in blue) corresponding to the full-length 361-bp transcript is shown.
